# CombiROC: an interactive web tool for selecting accurate marker combinations of omics data

**DOI:** 10.1038/srep45477

**Published:** 2017-03-30

**Authors:** Saveria Mazzara, Riccardo L. Rossi, Renata Grifantini, Simone Donizetti, Sergio Abrignani, Mauro Bombaci

**Affiliations:** 1 Istituto Nazionale Genetica Molecolare “Romeo ed Enrica Invernizzi”, Milan, 20122, Italy; 2 DISSCO, Department of Clinical Sciences and Community Health, University of Milan, Milan,20122 Italy

## Abstract

Diagnostic accuracy can be improved considerably by combining multiple markers, whose performance in identifying diseased subjects is usually assessed via receiver operating characteristic (ROC) curves. The selection of multimarker signatures is a complicated process that requires integration of data signatures with sophisticated statistical methods. We developed a user-friendly tool, called CombiROC, to help researchers accurately determine optimal markers combinations from diverse omics methods. With CombiROC data from different domains, such as proteomics and transcriptomics, can be analyzed using sensitivity/specificity filters: the number of candidate marker panels rising from combinatorial analysis is easily optimized bypassing limitations imposed by the nature of different experimental approaches. Leaving to the user full control on initial selection stringency, CombiROC computes sensitivity and specificity for all markers combinations, performances of best combinations and ROC curves for automatic comparisons, all visualized in a graphic interface. CombiROC was designed without hard-coded thresholds, allowing a custom fit to each specific data: this dramatically reduces the computational burden and lowers the false negative rates given by fixed thresholds. The application was validated with published data, confirming the marker combination already originally described or even finding new ones. CombiROC is a novel tool for the scientific community freely available at http://CombiROC.eu.

Powerful biomarkers have become and will continue to be important tools in diagnostic, clinical and research settings. In the area of diagnostic medicine, a biomarker is often used as a tool to identify subjects with a disease, or at high risk of developing the disease. Moreover, it can be used to foresee the more likely outcome of the disease, monitor its progression and predict the response to a given therapy. Accurate markers are powerful tools to help clinicians in the choice of the most appropriate treatment, ultimately improving a personalized patient management. In the last years the identification of disease-associated signatures has been accelerated by the use of high-throughput omics techniques^1^,^2^,^3^. Unfortunately, most of them could be too expensive to achieve the specificity and/or sensitivity needed for diagnostics and routine clinical practices. Moreover, it is now generally accepted that single markers do not achieve sufficient sensitivity and specificity for translation into diagnostic setting, thereby clinical decision-making process benefits from combining signatures to improve clinical performances: this trend clearly emerged over the past few years^4^,^5^,^6^,^7^,^8^. Literature provides various statistical modeling strategies to combine biomarkers among them, threshold-based^9^,^10^, logistic regression^11^,^12^ and tree-based^13^ methods are probably the most utilized whereas techniques such as Support Vector Machines^14^ represent helpful tools in many high-dimensional problems. Even if quite a number of statistical methods for combining biomarkers exists, their application in clinical environment is still a prerogative of analytically skilled researchers mainly due to the difficulty to extrapolate simplified, standardized and interpretable result from these complex statistical strategies^15^,^16^,^17^. Moreover, confirmation analyses are necessary to evaluate the performance of candidate biomarker panels in order to avoid the risk of over-fitting, which request to conduct a validation step on an independent cohort, or alternatively, by means of cross validation or bootstrapping^18^,^19^.

In this study we describe CombiROC, an easy-to-use tool implemented as a web application to accurately determine optimal combinations of markers from diverse complex omics data. CombiROC takes advantage of the combinatorial analysis and ROC curves; although these methods are being used in medicine and other areas for many decades, they generally lack an easy-to-use interface that researchers without programming skills could use to analyze data and to make plots. CombiROC stems from our previous screening for multi-marker signatures for Autoimmune Hepatitis (AIH)^20^. In this effort we realized that determining the best threshold values for a clinical diagnosis separating “abnormal” from “normal” samples is far from straightforward. With CombiROC it is possible to select the optimal markers combination(s) and obtain an immediate visual feedback, such as graph plotting and ROC curves, through a simple and interactive, yet statistically rigorous, workflow.

We validated the CombiROC method with independent datasets from both the screening and validation phases of a study on myotonic dystrophy type 1 patients^21^, that we treated without any a-priori knowledge thus processing them in a unbiased way. Moreover, by applying CombiROC to published data from proteomics^20^,^22^ and transcriptomics^23^, we were able to reproduce the markers combinations described in those studies, as well as to find better ones, in terms of sensitivity and specificity. Overall, CombiROC has unique and unprecedented properties: it uses an intuitive interface, it can be accessed by any browser, and it is freely available at http://combiroc.eu. A full online tutorial can also be found, allowing users to practice with pre-loaded real datasets.

## Results

### The CombiROC WEB APP: the workflow

**Figure 1:**
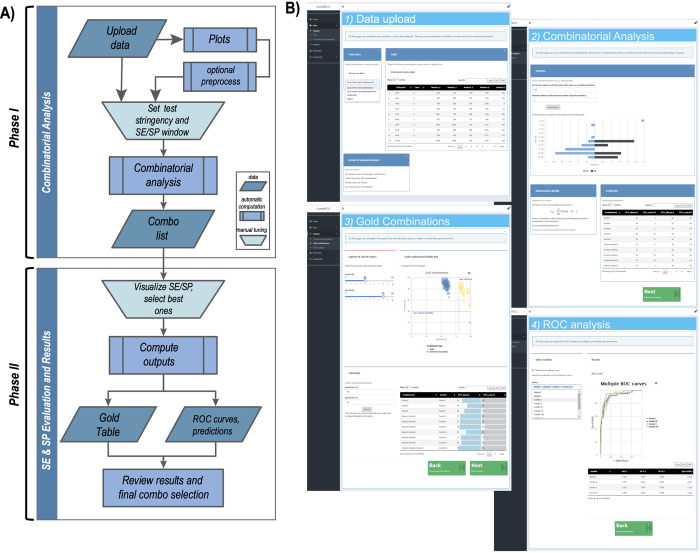
Sequential flowchart, web interface and output of CombiROC. (**A**) The flowchart is divided in two main phases. In the first one, multi markers profiling data are uploaded, plotted and users can define the stringency of their test. Finally the combinatorial analysis is carried on and a list of markers and combinations of markers (Combo list) is obtained. In the second phase thresholds on SE and SP can be visually explored and interactively adjusted observing how many marker combinations remain after imposing the cutoffs; the application then computes the outputs and automatically selects the best combinations (displayed in the Gold table)and pre-generates their ROC curves. (**B**) Schematic representation of the main steps of an output page. After uploaded data (*1*), a combinatorial analysis (*2*) is computed and the best combination of markers is displayed (*3*). Finally, markers either alone or in combination are analyzed by ROC curve (*4*).

CombiROC, delivers a simple workflow to help researchers in selecting the optimal combinations of markers and providing an easy-to-use interface. The workflow can be split conceptually in two main sequential phases (Fig. 1). The first one is the combinatorial analysis which takes as input the data corresponding to the two classes/categories of samples to be compared in a pairwise manner (such as healthy and disease, treated and untreated) and computes all the possible combinations of markers given a set of constraints and optional data transformation. The second phase consists in the computation and evaluation of sensitivity (SE) and specificity (SP) of all markers combinations and the subsequent candidates’ combinations selection and Receiving Operator Curves (ROC) calculation, up to the selection of the final and best performing group. Details of all specific steps are described.

#### Phase 1. Data uploading and set up

Data from profiling results of two classes or categories to be compared can be uploaded in text format, which may include comma, tab or semicolon separated text files (see example in Supplementary Fig. 1 and in the online tutorial). After dataset uploading and automatic display of a descriptive statistics (Supplementary Fig. 2), the user is given the option to review data and sample details (i.e. number of samples, markers and categories, data value’s type, and missing values), to confirm their quality. Box plot and marker profile plot are also automatically displayed and help users evaluate the overall distribution of data. Box plot and its descriptive statistics is used to visualize the distribution of single markers values across the samples (i.e. patients) while marker profile plot displays the signal intensity of each single sample for each marker. These plots can be easily customized to visualize keys details embedded in the data structure.

To facilitate data analysis and model interpretation, the application offers the option to pre-process uploaded data using natural logarithmic transformation and/or data scaling (including unit variance scaling/autoscaling in which the scaling factor is the standard deviation, and pareto scaling in which the scaling factor is the square root of the standard deviation); these are popular methods in OMICs, whose use depends on the nature of data (e.g. cell counts, fluorescence intensities, number of genes)^24^,^25^. Moreover, user’s uploading data errors are detected and displayed by a warning message.

Then test stringency needs to be set: in this key step the test’s signal cutoff and the minimum number of positive features above the cutoff can be modified and should be dictated by the specific nature of the technological platform used for markers detection. The aim of this step is to submit to the combinatorial analysis that follows only those markers whose signal was positively detected; each user is responsible for the assessment and use of the specific detection limits since this limit depends on the detection method used to generate the data.

#### Phase 2. Combinatorial analysis and selection of “gold combinations”

Generation of the marker combinations based on SE and SP filters. Once the test signal and minimum features cutoffs are set, the application performs a combinatorial analysis and calculates SE and SP scoring for each marker combinations, interpreted as the probability (expressed as a percentage) that the assay data will be positive when the disease is present and as the probability that assay data will be negative when the disease is absent.

**Figure 2:**
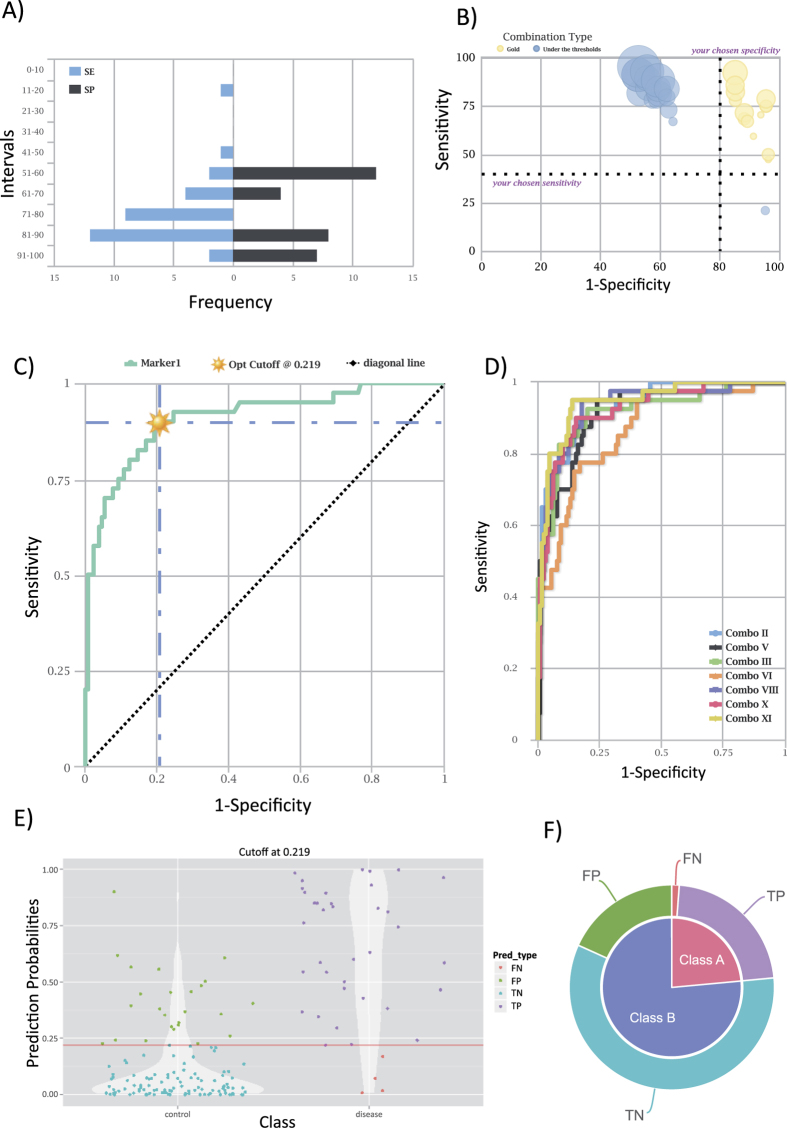
Representation of the main steps of the analysis process. (**A**) Distribution of the specificity (SE) and sensitivity (SP) of positive markers, automatically determined taking into account all the features (markers) that are above the set stringency values. X-axis shows the number of each positive feature falling into a specific interval of SE and SP percent frequency to be used as filters of the subsequent steps. (**B**) Combinatorial analysis of marker profiles. Interactive bubble chart showing the sensitivity (Y-axis) and specificity (X-axis) of all the feature (marker) combinations; the size of the bubbles is proportional to the number of markers in the combo. Yellow bubbles represent the marker combos with SE and SP values above the manually chosen thresholds, which will be used in the ROC curve calculation step. (**C** and **D**) graphic representation of ROC curve for single marker or for comparing multiple marker and their combinations selected in the Gold table, respectively; in C the plot also displays the identity line (diagonal) and the point of optimal cut-off; (**E**) Violin plot showing the probability density of the data for the two compared classes, dependent on the previously obtained optimal cutoff on the corresponding ROC curve (**F**) Pie chart showing the fractions of false predictions (false positive, FP, and false negative, FN) as well as true predictions (true positive, TP, and true negative, TN) relative to the two classes.

Ideally a medical test should have high values of both SE and SP, so marker combinations with small values are considered to be of negligible importance from a clinical point of view. The distribution of these metrics is shown in Fig. 2A, where SE and SP intervals in percent are plotted against the frequency of each positive combination: such a view allows to select the optimal cutoff for these metrics optimizing the number of potentially interesting marker panels.

#### Selection of the gold marker combinations by choosing the best threshold for SP and SE filters

Each marker and combination thereof are visualized by an interactive bubble plot (Fig. 2B), in which the bubble size is proportional to the number of markers of each combo. All markers and combinations are depicted by bubbles in the SE and SP space allowing the user to visually inspect their performance: based on what is displayed the user can raise SE and SP threshold in order to exclude the worst performing ones and to keep the best ones. Combinations that do not bypass the SE and SP thresholds set are depicted as blue bubbles (the “under the thresholds” combos), whereas good combination are visible as yellow bubbles (the “Gold” combos). Since there are no limitations to the number of combinations that could be managed by the analysis, in theory, one could even keep all the possible combinations (leaving SE and SP thresholds set at zero) and bring them to the next steps. However, for an efficient biomarker selection it’s better to keep the best performing combinations only. Once the minimum SE and SP values are finally chosen and confirmed, the “gold” combinations are listed in tabular form along with the relative SE/SP values and single markers composition (Supplementary Fig. 3).

### ROC analysis and performance analysis for the best marker panels

ROC curves of markers and combinations selected in the previous step are calculated with all key parameters (Area Under the Curve, AUC and Optimal cutoff) and graphically visualized (Fig. 2C,D). Prediction probabilities are also evaluated using a graphical representation, such as violin plots and pie charts.

All four possible categories (False Negative, FN; False Positives, FP; True Negative, TN; True Positive, TP) are displayed through a violin plot which help visualize the proportion of samples falling in the four possible quadrants, especially in those of the TN and TP predicted categories, in order to evaluate the goodness of the underlying markers (Fig. 2E). The pie chart shows the very same information in a different way. In this plot it can be easily visualized which fraction of false predictions (false positive or false negative) in each class (class A/B, disease/healthy) as opposed on how big is the fraction of true predictions and inside the total fraction of markers in each class. Obviously the smaller the false predictions fractions, the better performing is the marker or the combination (Fig. 2F).

### Validation of the CombiROC method

**Figure 3:**
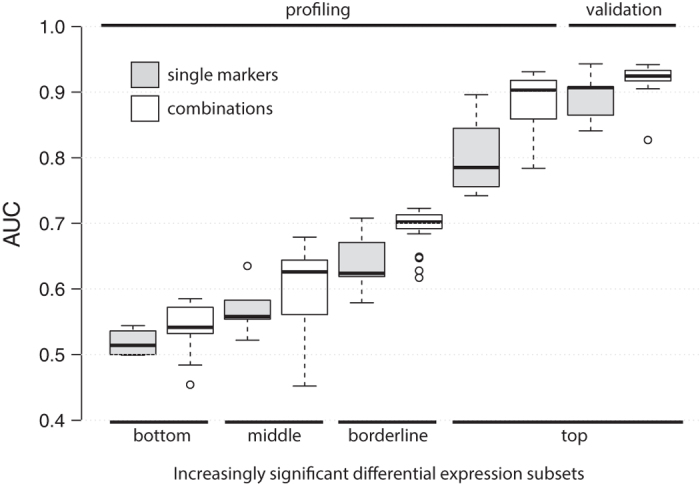
AUC values distributions of markers and combinations across validation subsets. Distributions of Area Under the Curve (AUC) values as determined by CombiROC for the four microRNA profiling subsets across their significance range (see lower horizontal black bars) of the differential expression analysis: the 5 less significant microRNAs (bottom), the 5 non significant microRNAs in the middle range (middle), the 5 microRNAs just below the significance cutoff (borderline), the 5 most significant microRNAs (top). Gray boxes show the distribution of the AUC values of the 5 single microRNAs, white boxes show the distribution of all 26 possible combinations of the previous 5 single microRNAs. Plot’s upper horizontal black bars distinguish data of the profiling dataset (the 8 leftmost box plots) from data of the validation dataset (the 2 rightmost box plots).

In order to validate the method we used the complete data from a study searching biomarkers among plasma microRNAs in myotonic dystrophy type 1 (DM1) patients^21^. Starting from the available raw screening data of microRNAs detected by high throughput RTqPCR from 50 plasma samples (24 patients, 26 healthy donors) and following the method described in the paper, we performed a *de-novo* differential analysis obtaining a ranking of all detectable microRNAs and a final signature of significantly differentially expressed microRNAs. We then asked whether the top microRNAs in the selected signature performed as biomarkers according to the CombiROC procedure when compared to non significant microRNAs, and how CombiROC would differentiate these microRNAs according to their calculated significance. Since *n* features (genes, microRNAs, proteins) can be combined in (2^n^ − 1) ways, just 100 features would lead to 1.26 ^∗^10^30^ combinations. Thus, in order to obtain manageable yet informative numbers, we sampled 4 different smaller sub datasets of 5 microRNAs each across the significance-ranked profiling data results. In particular, we selected *i)* the top 5 significant microRNAs (“top” subset), *ii)* the 5 microRNAs just below the significance cutoff thresholds of pValue = 0.05 (“borderline” subset), *iii)* 5 microRNAs in the middle of the list (“middle” subset) and finally *iv)* the less significant microRNAs namely the 5 microRNAs with the highest p-Values (“bottom” subset) (Supplementary Table 1). Each subset of 5 microRNAs, with their expression values in the 50 samples was then used as CombiROC input. The AUC values for the 5 single markers and their 26 possible combinations were determinate by CombiROC (Supplementary Table 2) and their distributions compared between single markers and combinations across the 4 subsets. The AUC distributions of less significant subsets were lower than those from significant subsets (with lower p-Value microRNAs), confirming the CombiROC ability to quantitatively discriminate and correctly ranking potential biomarkers in a signature (Fig. 3). Moreover, the combinations determined with CombiROC showed in all cases bigger AUCs (white boxes in Fig. 3) compared to those of the corresponding single microRNAs (grey boxes in Fig. 3), even for microRNAs well below the significance cutoff (p-Value = 0.05). Top hits were validated by Perfetti and colleagues by single qPCR assays, without multiplexing, extending the profiling to all recruited patients and controls for a total of 74 samples^21^: using the expression values of the top 5 microRNAs from the dataset of this independent validation cohort we determined with CombiROC the AUC distributions and further confirmed the trend observed in the profiling dataset (Fig. 3, two righmost boxplots). Finally we also showed that CombiROC is able to reproduce the very same AUC value and ranking of all top 9 microRNAs originally determined by the authors (miR-133a, miR-140-3p, miR-191, miR-193b, miR-27b, miR-454, miR574-3p, miR-885-5p, miR-886-5p: Supplementary Fig. 4, from “marker0” to “marker8” respectively), with a complete correspondence of AUC values and ROC curves.

### CombiROC facilitates and confirms the selection of protein biomarkers combinations for autoimmune hepatitis

We then wanted to show CombiROC’s versatility using other datasets derived from two different studies, one focus on proteins and another on microRNAs potential biomarkers. Results from these analyses were also used to build the demo-datasets and tutorial available online to illustrate the functionality of CombiROC.

**Figure 4:**
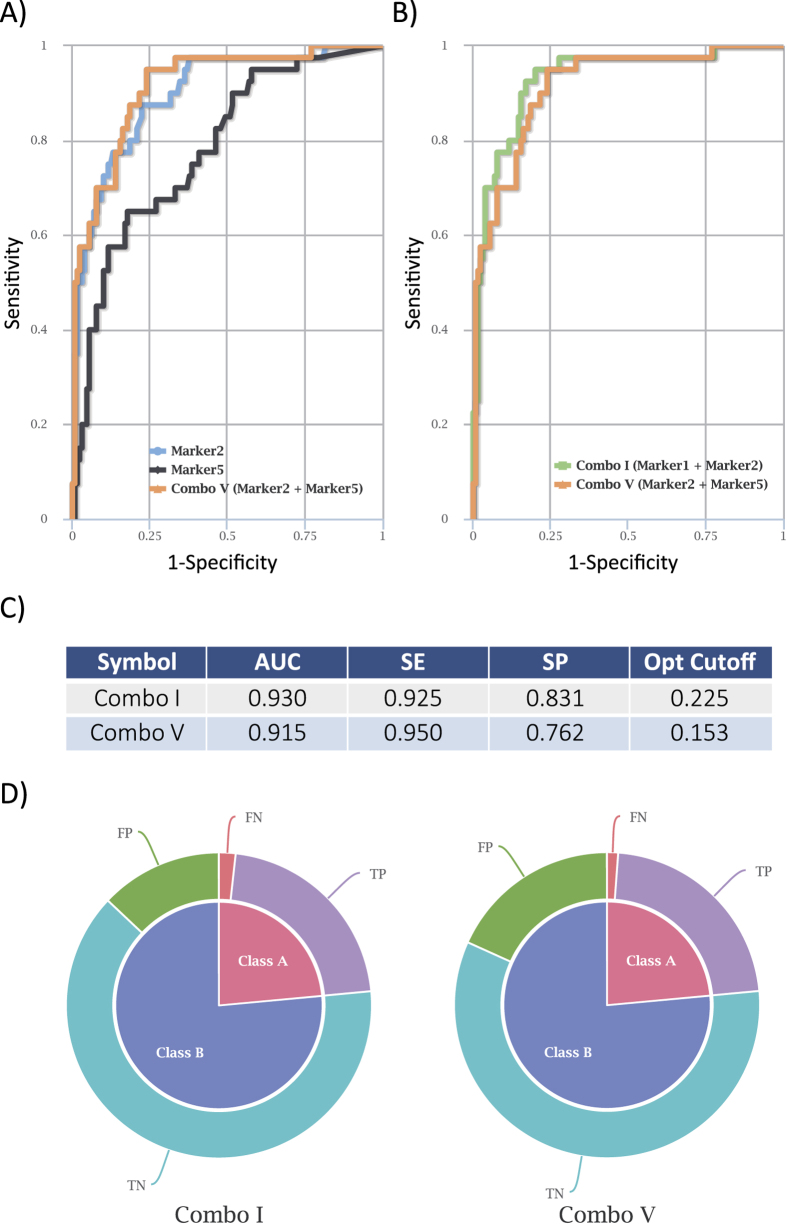
Combinations of autoimmune hepatitis protein markers identified by CombiROC. (**A**) ROC curve comparative plot of UNQ9419 (marker2), CHAD (marker5) and their combination (Combo V). (**B**) ROC curve comparison of Combo V and the better performing Combo I (marker 1, IL4R + marker2, UNQ9419). (**C**) Table summarizing relevant parameters of all compared curves. (**D**) Pie charts showing the fraction of predictions (FN: false negative; FP; false positives; TN; true negative; TP; true positive).

As a first test, we employed CombiROC to re-analyze proteomics data published by our group. We recently discovered a 5-biomarker signatures, IL4R, AQA598, RSPO3, CHAD and UNQ9419 for AIH, by serological screening of patients with AIH and healthy donors (HD) on a protein microarray representing 1626 human proteins^20^,^22^, and asked if any different marker combination had equal or higher performance. The original data of this study were uploaded in CombiROC: they consists of 170 samples (40 AIH patients and 130 healthy controls) with corresponding levels of the 5 identified biomarker proteins (see Data table 1). Serum fluorescence signals were scored as positive when at least 1 marker on the protein array was detected with a minimum fluorescence intensity higher than mean negative control signal plus three times the standard deviation (in our case equal to 450 fluorescence units). With such parameters, the-combinatorial analysis step generated a list of 31 markers combinations. Setting the sensitivity and specificity filters to 40% and 80%, respectively, 14 out of 31 marker combinations were selected, including 3 individual markers. The combination named “ComboV” given by UNQ9419 and CHAD was among the top ones according to their AUC, SE and SP values (Fig. 4A) confirming what shown in the AIH study^22^. Moreover, a comparative analysis of the ROC curves generated automatically for each combination upon setting of the desired parameters, allowed to identify at least one additional combination with better accuracy values (ComboI) formed by IL4R and UNQ9419 (Fig. 4B). A visual representation of area under curve (AUC), SE, SP and cutoff values, as well as false positive and false negative rates automatically generated for each marker/marker combination (see the relevant ones in Fig. 4C), allowed to quickly evaluate their impact and variation across selected combinations (Fig. 4D).

### CombiROC identifies accurate microRNA markers combinations for primary central nervous system lymphoma

**Figure 5:**
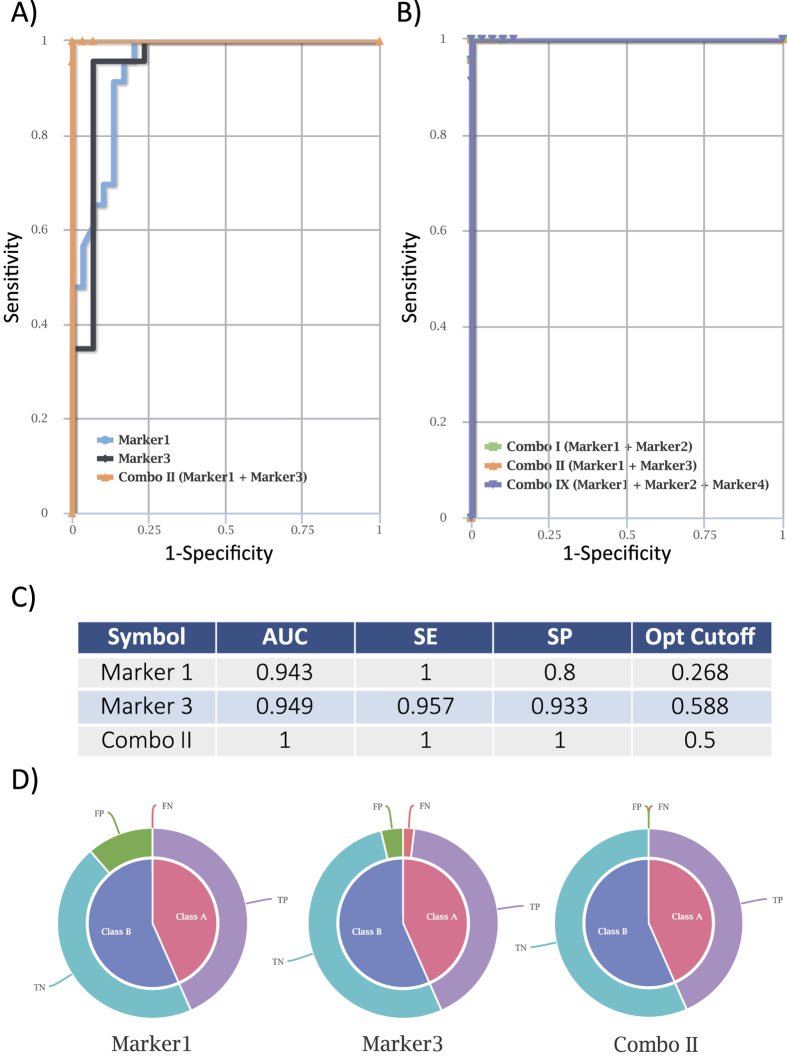
microRNA markers of primary central nervous system lymphoma identified by CombiROC. (**A**) ROC curve comparative plot of of miR-21 (marker1), miR–92a (marker3) and combination thereof (Combo II) as obtained using the CombiROC application on Baraniskin *et al*. data^23^. (**B**) ROC curve comparison plot showing Combo II, and the equally performing Combo I (marker1,miR21 + marker2, miR-19b) and Combo IX (miR-21 + miR-19b + miR-15b, marker4) are equally performing. (**C**) Tabular view displaying parameters of miR-21 and miR–92a and their combination (Combo II). (**D**) Pie charts showing the fraction of predictions (FN: false negative; FP; false positives; TN; true negative; TP; true positive).

We further challenged CombiROC reproducing combinatorial analysis from another work whose detailed analysis methods and original datasets at the raw, unprocessed level were publicly available and a ROC analysis was performed. To this aim, we selected microRNA transcriptomics data published by Baraniskin and colleagues, which led to the identification of a 6-microRNAs biomarker signature for the diagnosis of Primary Central Nervous System Lymphoma (PCNSL)^23^. Original data from this study were obtained from 53 cerebrospinal fluid samples (23 patients with PCNSL and 30 control individuals) relative to the 6 microRNAs (miR-21, miR-19b, miR-92a, miR-15b, miR-106b, miR-204). Such data, retrievable from Data table 2, were formatted and uploaded to CombiROC application. To preserve their original structure, data were not subjected to any transformation step. A “test’s cutoff signal” value of 8 and a “minimum number of features” of 1 were chosen as inclusion criteria for the Combinatorial Analysis step: these values were independently chosen and may not be the same used by the authors, but were chosen as those yielding the highest sensitivity and specificity values in with a reasonable frequency intervals to perform downstream analysis. All the 63 possible combinations of the above 6 microRNAs/markers were obtained in the Combo List box. With a sensitivity and specificity at least equal to 65% and 70% respectively, 18 of these combinations remained including 2 single markers (miR-21 and miR-92a), which were also scored as the top two candidates in the original work^23^. CombiROC generated ROC curves of single microRNAs (such as for miR-21 and miR–92a) very similar to the published ones, in terms of AUC, SE and SP values^23^. Remarkably, a marker combo with even higher sensitivity and specificity was also identified (Combo II, composed of miR-21, and miR-92a) (Fig. 5A,C,D). Moreover, other highly performing microRNA combinations, not described in the original paper, could be identified by CombiROC, including different markers (e.g. Combo II: miR-21 and miR-19b; Combo IX: miR-21, miR-19b and miR-15b) (Fig. 5B). CombiROC analysis revealed a redundancy of highly performing markers combinations, often composed of markers that if individually considered would not achieve sufficient specificity and sensitivity to pass the initial screening criteria.

The possibility to select combinations which are redundant in performance but different in composition, is a potential advantage in terms of flexibility of a diagnostic test that could be more robustly designed deploying parallel tests with different - but equally performing - combinations of markers, thus minimizing any detection or technical failure.

## Methods

### Development environment

The core of CombiROC is written in R^26^ powered by the web application framework Shiny^27^ hosted on a Linux server. The data manipulation and the data analysis are executed using R-scripts, which relies either on custom functions or CRAN packages including *shinydashboard*, to create easily attractive dashboards; *DT*, to render data objects as HTML tables providing advanced interaction controls; *gtools* for creating combinations; *rmarkdown* for generating dynamic documents; *ggplot2*for creating elegant and complex plots; *ineq, e1071* and *stringr*, for data manipulation; *pROC* and *cvAUC* for ROC analysis. During the working session, the R tasks are executed as back-end processes not requiring the R knowledge. Moreover, *Highcharts JS* library^28^ is used at the client side to offer interactive charts, from multitouch zooming to touch-friendly tooltips. The user data are held temporarily and discarded as soon as the session terminates and none of the data is stored remotely on any server.

### Test stringency and combinatorial analysis

Individual biomarkers and the biomarker combinations are examined using a user-defined test stringency*D* = {*thr, minf*}, according to the specific nature of the user experiment (the test), to classify samples as positive or negative cases where *thr* denotes the cutoff above which the features’ values are considered positive (the “test’s signal cutoff”) whereas *minf* represents the minimum number of features that need to reach the previously cutoff within the feature panel. Successively, all possible combinations, both as single marker and combination markers, were generated; given N biomarkers, the total number of enumerable combination is:


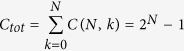


where N is the total number of items (the total biomarkers) and k is the number of components in the combination. Given the collection of all the combinations of a dataset, it was mandatory to identify a combination score related to the generated combinations. At this point, each biomarker (single or combination) is assigned a binary result (a 1 or a 0) based upon whether the detected level of the biomarker in the sample exceeds the signal cutoff, *thr,* so samples with one biomarker value exceeding the signal cutoff, *thr*, were classified as positive whereas when a combination of biomarkers was used, at least *minf* biomarkers exceeding the *thr* value was considered to classify the sample as positive test.

### The sensitivity and specificity space and the Gold combinations

For each of the combinations satisfying the hard thresholds, the Sensitivity (*SE*) and Specificity (*SP*) filters, interpreted in terms of recognition frequency, are computed. SE is defined as the true positive rate in percentage of your sample whereas SP is defined as the true negative rate in control class in percentage. The sensitivity and specificity are reported either as a numeric value or as histogram plot. This allows to evaluate the intervals from which the best SP/SE values will be chosen, and on which markers’ “*Gold Combinations*” will be calculated in further steps of the analysis.

### ROC curve

The discrimination power of biomarker panels is assessed by ROC curve analysis which combines SE and SP of a given marker for diagnostic test evaluation which ranges from 0.5 (no discriminating power) to 1.0 (complete separation). In a ROC curve plot, the true positive rate, “sensitivity” (y-axis), is plotted as function of the false positive rate, “1 – specificity” (x-axis). Large “y” values on the ROC curve plot correspond to higher sensitivity, while small “x” values correspond to higher specificity. The shape of the curve depicts the combinatorial variation of these two important parameters. Below the ROC curve you will be able to see the AUC (Area Under the Curve), SE, SP and optimal cutoff numerical values of the analyzed combo in tabular form. The results are reported in fractions from 0 to 1. A diagonal line of identity is reported such as the point of optimal cut-off.

### Predictions

The “Predictions” section is visualized by violin plot and pie chart. The violin plot is a combination of a box plot and a kernel density plot showing the “probability density” of the data at different values. Prediction probabilities are plotted for both classes (class A and B, disease/healthy, treated/untreated) according to the previously obtained optimal cutoff on the corresponding ROC curves.

### Cross-validation and permutation test

To give an estimate of the panel performance in an unbiased manner a cross-validation (CV) procedure was introduced; without proper validation testing, strong biases, such overfitting, undermine the robustness of a classification model.

A single CV procedure allowed us to randomly generate the training and test partitions from the original dataset, in this way ROC curve of a biomarker panel based on both the training and the test set is performed. To give reliable estimate of model performance, multiple rounds of CV are performed using different test sets; so, a family of ROC curves are generated and the performance results are avaraged over the rounds. A 10-fold CV strategy was used to compare the different models generated.

One limitation of CV is that it provides over-optimistic results, so in order to give a measure of the statistical significance of the AUC value and to test the model for over fitting due to chance correlation a permutation test was introduced. Thus, only the Y-block (class labels) was randomly reordered while the X-block (expression matrix) was left intact. By repeating this procedure N times, a model is fitted to the new Y-data and new estimates of AUC values were calculated^25^. In this way, a reference distributions of AUC is obtained, useful for appraising the statistical significance of such parameter; if “real” values are found outside such distributions this is a sign of high validity of model^25^.

### Datasets

Three real experimental datasets were used to test its applicability to different “omics” fields and to validate and illustrate the functionality of CombiROC.

Raw data on plasma microRNAs in myotonic dystrophy type 1 (DM1) patients were used for method’s validation. Datasets consist of 50 samples (26 controls and 24 DM1 patients) in a high through-put RTqPCR screening phase and 72 samples (36 controls and 36 DM1 patients) in a validation phase by single RTqPCR assays^21^ (personal communication). To rank microRNAs as potential biomarkers in DM1 patients. we considered the detected microRNAs as described in the original paper^21^. Raw data from RTqPCR (raw CTs) were processed with the Delta Ct comparative method, using global median normalization. Normalized data were then linearized and subjected to t-test statistic with an overall alpha cutoff of 0.05.

#### Proteomics data

Data on autoimmune hepatitis (AIH)^22^, (see Data table 1) relative to 170 samples (40 AIH patients and 130 healthy controls) and 5 features (protein IDs: IL4R, UNQ9419, A0A598, RSPO3, CHAD, marked in the data table header with the labels *marker1* to *marker5* respectively).

#### Transcriptomics data

Data on microRNA biomarkers for the diagnosis of primary central nervous system lymphoma (PCNSL)^23^, see Data Table 2, refer to expression level in cerebrospinal fluid are extrapolated from Supplemental Tables 1 and 2, and are originally obtained from 53 samples (23 patients with PCNSL and 30 control individuals) and relative to 6 microRNAs (*miR-21, miR-19b, miR-92a, miR-15b, miR-106b, miR-204*) selected by the authors as candidate markers. Data of the above mentioned microRNAs were formatted and uploaded to CombiROC application; in the “Demo data, transcriptomics” the microRNAs *miR-21, miR-19b, miR-92a, miR-15b, miR-106b, miR-204* were marked with the labels from *marker1* to *marker6* respectively. In order to preserve their original structure, PCNLS data were taken as is and not subjected to any optional transformation step.

## Discussion

The investigation, discovery and optimization of biomarkers in the context of translational medicine is of fundamental importance for accurate diagnosis and monitoring of disease, predict the most likely outcome and the response to a given therapy. It is now well accepted that single markers may not be sufficiently accurate, while multi-marker combinations can achieve significant specificity and sensitivity values. Indeed, a number of assays based on multiple markers is already commercialized and used in clinic (e.g. Prometheus Crohn’s Prognostic Test, Prometheus Ibd Serology 7, Prometheus Celiac Serology - https://www.prometheuslabs.com/Products/Default.aspx; Mammaprint 70-Gene Breast Cancer Recurrence Assay^29^, - http://www.agendia.com/healthcare-professionals/breast-cancer/mammaprint/). Nevertheless, the demand of novel markers signatures associated to life-threatening human diseases is still very high. Many research and clinic laboratories are putting their efforts to address this need by exploiting various OMICs technologies. However, the identification of multi-marker signatures is often time-consuming and labor-intensive for common research and clinical laboratories, and the use of the needed bioinformatics and statistical methods requires skilled scientists making this process of not trivial application.

During the discovery of biomarker signatures in our work on autoimmune liver diseases^20^,^22^, we had to tackle the difficulty of efficient and robust computational selection of markers combinations. Thereby, we decided to pursue the development of a tool combining a simple user interface, robust statistical underlying methods, and applicability to data from different omics fields, thus allowing to bypass computational difficulties of combinatorial analysis and biomarker selection. This tool was first intended for internal use, then scaled up for the broader community in the form of a web application.

In this paper we presented CombiROC web application, written in R with the Shiny application framework. CombiROC is a user-friendly, reliable analytical tool to support researches in the selection of optimal marker combination(s) through a simple and interactive workflow. Through its interactive interface, it allows users to make choices based on evaluation of all possible markers, double-filter scoring on sensitivity and specificity, selection of best-performing combinations, and visualization of their receiver operating characteristic (ROC) curves.

CombiROC is a tool devoted to the ranking and selection of potential biomarkers from signatures of any kind of feature (i.e. genes, proteins, non coding RNAs). Prior to submission to CombiROC, any given initial screening dataset is required to undergo a data reduction process typically performed with differential expression analysis or similar methods. Given its combinatorial nature, CombiROC is not designed to directly analyze profiling datasets since this would lead to unmanageable number of combinations. In fact, any *n* features (genes, microRNAs, proteins) can be combined in (2^n^ − 1) ways, thus 5 features lead to 31 combinations, 10 features to 1032 and 100 features would lead to 1.27 * 10^30^ combinations. We believe a reasonable number of initial potential biomarkers to analyze with CombiROC is usually below 10. CombiROC is fully focused on ROC-curve centered analyses bases on a 2 by 2 contingency table, thus it handles two-class problems such as the most common case in which pathological samples need to be distinguished from healthy ones. The two classes analyzed can indeed belong to two different stage/grades of a disease (as in disease subtyping) but more complicated subtyping would be a multi-class problem and it should be analytically treated differently.

The test stringency can be manually adjusted (i.e., the test signal cutoff and minimum number of positive features that are above the cutoff). CombiROC then determines sensitivity and specificity of all possible marker combinations. Picking a threshold for these metrics is not straightforward as their absolute values depends on the specific nature of the experiment that generated the data and on the aim of the user. One of the most evident added value of our web application is the possibility to select the best combinations interactively, by exploring the sensitivity/specificity space with immediate visual feedback thereby evaluating any taken tradeoff. Users set their final thresholds and see identity and performance details of the optimal combinations. ROC, prediction, and performance analyses are automatically performed, allowing comparison of combinations. This guarantees an optimal classification, and is better suited to the non-normally distributed data commonly found in clinical studies, where the last increments may not be as significant as the first ones. CombiROC automatically manages all of the above using a recognition frequency analysis encompassing the limitations imposed by the nature of different biomarker panels. Graph plotting are an important support of data analysis, and many webtools are dedicated to this^30^,^31^, but data visualization is not the main function of our application: CombiROC uses graphs and visual feedback in order to provide a complete computational workflow, aimed at analyzing data by a threshold based method and optimizing interesting panels with combinatorial analysis.

An important part of our study was dedicated to the validation of CombiROC method with data from transcriptomics^21^, and to show its versatility in finding new marker combinations with proteomics^22^ and transcriptomics^23^ studies. By applying CombiROC analysis to these real datasets, we were able to rapidly confirm the performance of the published markers and we also found multi-marker combinations that outperformed single markers. Moreover, some of the high-value multi-markers combinations include markers not ranking among the top performing ones, which would have been eliminated or disregarded by strategies lacking a combinatorial analysis phase. This aspect is an important functional advantage of CombiROC, as it lowers the risk of missing important markers. Indeed, the possibility of flanking clinically acknowledged biomarkers with equally or even better performing combinations of independent markers could provide a more robust rationale for diagnostic decision making.

Overall, we believe the CombiROC application is a valuable tool mainly due to the following reasons: 1) it is a free tool that allows to perform robust combinatorial analyses without a specific training; 2) it allows the users to interactively intervene on the choice of thresholds, thus dramatically reducing the computational burden without sacrificing the statistical soundness; 3) it makes easier to analyze panels of biomarkers lowering false negative rate given by fixed thresholds.

Thanks to these advantages and ease-of use, CombiROC specifically alleviates the lack of expertise providing a fast, easy yet rigorous tool, and contributing to decrease costs to laboratory practice.

In addition to these technical and practical considerations, a comment is deserved on the usefulness of CombiROC to address so far unsolved biological questions. Indeed, combinations of multiple markers may suggest the existence of functional, physical or metabolic relations among single markers composing them. Although not specifically investigated in this study, we believe that CombiROC could help provide new hints in our knowledge of human diseases.

Future prospects of CombiROC foresee its applicability to integration of multi-OMICs data. Indeed, multivariate discovery approaches represent the natural evolution of this application, which enable combinatorial analysis of datasets generated by different OMICs technologies (e.g. gene vs protein microarrays, miRNA vs single reaction monitoring experiments, etc). This further development is in line with the recent directive of public funding agencies (e.g. Horizon 2020 calls) promoting the integration of multiple OMICs to find and validate the most accurate marker combinations.

## Additional Information

**How to cite this article:** Mazzara, S. *et al*. CombiROC: an interactive web tool for selecting accurate marker combinations of omics data. *Sci. Rep.* **7**, 45477; doi: 10.1038/srep45477 (2017).

**Publisher's note:** Springer Nature remains neutral with regard to jurisdictional claims in published maps and institutional affiliations.

## Supplementary Material

Supplementary Information
