# Supplementary material for: CombiROC: an interactive web tool for selecting accurate marker combinations of omics data
Source: Sci Rep. 2017 Mar 30;7:45477. doi: 10.1038/srep45477 (PMC5371980; doi:10.1038/srep45477)
Supplement: Supplementary Information [file srep45477-s1.pdf]

CombiROC: an interactive web tool for selecting accurate marker combinations of omics data

1. Saveria Mazzara
2. Riccardo L. Rossi
3. Renata Grifantini
4. Simone Donizetti
5. Sergio Abrignani
6. Mauro Bombaci

Supplementary information

Document includes:

Supplementary Figure 1

Supplementary Figure 2

Supplementary Figure 3

Supplementary Figure 4

Supplementary Table 1

Supplementary Table 2

Data table 1 - AIH proteomics

Data table 2 - PCNSL transcriptomics

Tutorial

SUPPLEMENTARY FIGURES

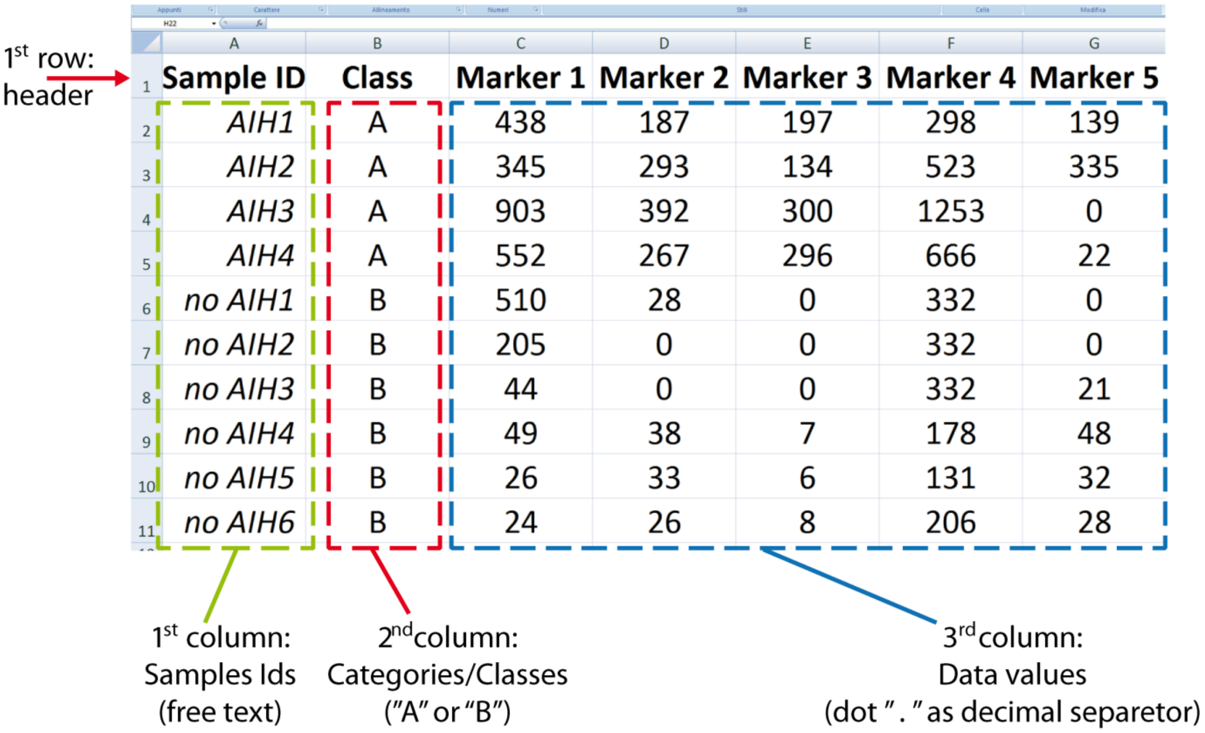

Figure S1. Representation of the table correctly formatted to be analysed by CombiROC. The first row can contain a header. The header of the *(i)* first column must be "Sample_ID", of the *(ii)* second column must be "Class", the header of columns from the *(iii)* third onward must be "Marker #", where "#" is a progressive integer (e.g. Marker1, Marker2, Marker3, *etc*.). The second column, dedicated to the classes/categories of samples (i.e. healthy and diseases; treated and untreated) may contain an arbitrary number of classes but only the first 2 classes will be considered for pair wise comparison.

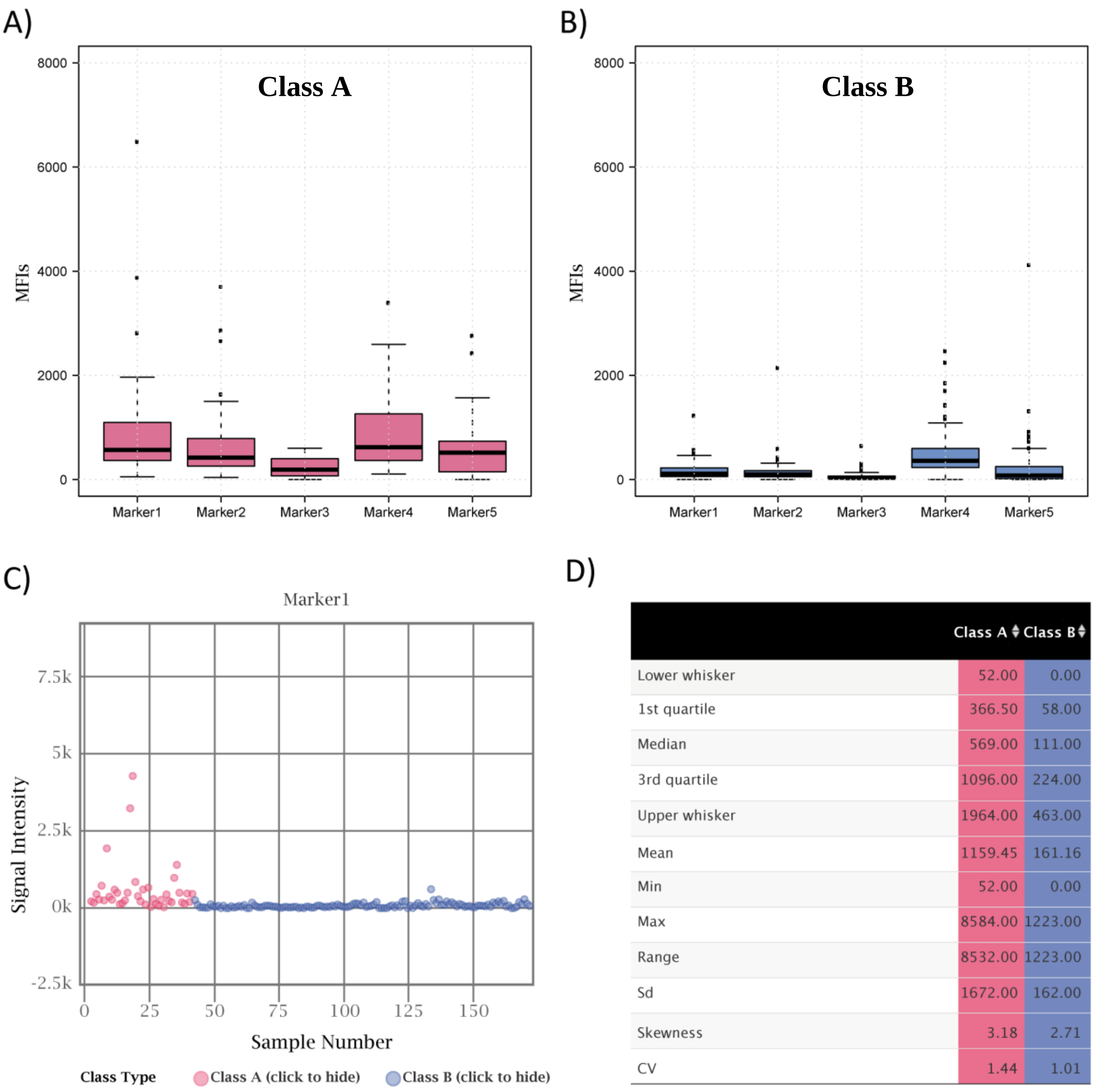

	Class A	Class B
Lower whisker	52.00	0.00
1st quartile	366.50	58.00
Median	569.00	111.00
3rd quartile	1096.00	224.00
Upper whisker	1964.00	463.00
Mean	1159.45	161.16
Min	52.00	0.00
Max	8584.00	1223.00
Range	8532.00	1223.00
Sd	1672.00	162.00
Skewness	3.18	2.71
CV	1.44	1.01

Figure S2. Graphic representation of the box plots and marker plot. (A-B) The box plot visualizes the distribution of single markers values across the two groups to be compared (Class A and Class B).; markers are visualized in each class and their distributions observed. **(C)** The marker plot displays the signal intensity of each single sample for each marker. **(D)** “Box plot statistics” table for both classes showing the distribution parameters, allowing the user to choose the best cutoff values for the subsequent step.

Show 10 entries Search: Copy CSV PDF

Combinations	Symbol	SE%_disease	SP%_control
Marker1	Marker1	65	95
Marker2	Marker2	48	98
Marker5	Marker5	58	88
Marker1-Marker2	Combo I	72	95
Marker1-Marker3	Combo II	72	95
Marker1-Marker5	Combo III	75	84
Marker2-Marker3	Combo IV	52	98
Marker2-Marker5	Combo V	70	87
Marker3-Marker5	Combo VI	68	88
Marker1-Marker2-Marker3	Combo VII	78	95

Showing 1 to 10 of 14 entries Previous 1 2 Next

Figure S3. List of potential candidate markers by CombiROC. The table displays the percentages of Specificity and Sensitivity corresponding to each marker or combination, based on the selected thresholds.

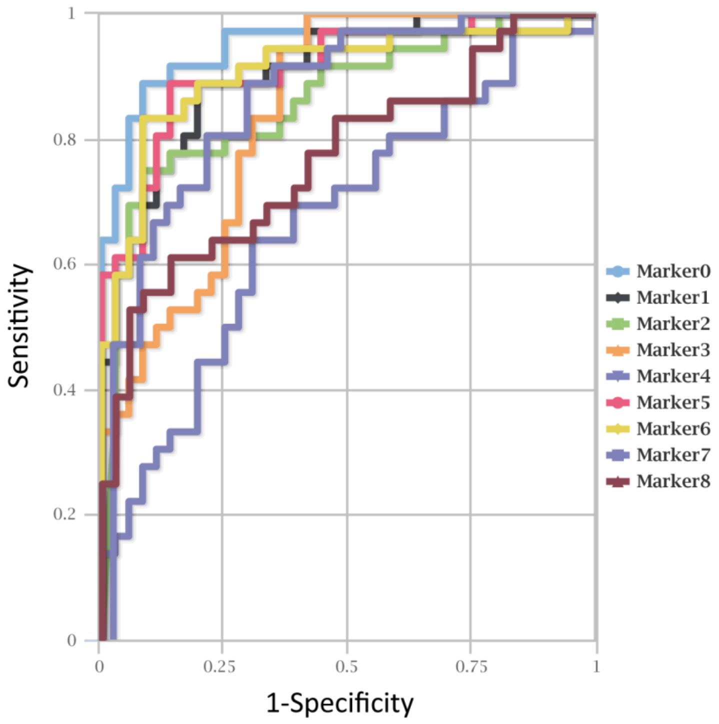

Symbol	AUC	SE	SP	Opt Cutoff
Marker 0	0.943	0.889	0.917	0.706
Marker 1	0.907	0.889	0.806	0.658
Marker 2	0.865	0.750	0.917	0.685
Marker 3	0.841	1	0.583	0.285
Marker 4	0.669	0.639	0.694	0.512
Marker 5	0.914	0.889	0.861	0.689
Marker 6	0.908	0.833	0.917	0.716
Marker 7	0.887	0.0.889	0.722	0.611
Marker 8	0.770	0.611	0.861	0.653

Figure S4. List of potential candidate markers selected from Perfetti *et al.* by CombiROC. *Top:* ROC curve illustrating sensitivity/specificity of miRNA identified from Perfetti and collaborators, here defined as Marker0 to Marker8 (miR-133a, miR-140-3p, miR-191, miR-193b, miR-27b, miR-454, miR574-3p, miR-885-5p, miR-886-5p, respectively), in discriminating between myotonic dystrophy type 1 (DM1) patients and CTR groups. *Bottom:* The table displays the AUC of SE and SP corresponding to each marker.

Supplementary Table 1

DEX	Subset	Detector	pVal (DEX analysis)	AUC (CombiROC)
significative	top	hsa-miR-133a-002246	1.55E-07	0.896
		hsa-miR-191-002299	8.45E-06	0.845
		hsa-miR-193b-002367	2.16E-04	0.785
		hsa-miR-574-3p-002349	3.29E-03	0.742
		hsa-miR-140-3p-002234	5.24E-03	0.756
non significative	borderline	hsa-miR-27a-000408	6.85E-02	0.708
		hsa-miR-146b-001097	7.85E-02	0.619
		hsa-let-7e-002406	8.88E-02	0.671
		hsa-miR-21-000397	1.05E-01	0.579
		hsa-miR-20a-000580	1.06E-01	0.624
	middle	hsa-miR-148a-000470	4.11E-01	0.635
		hsa-miR-145-002278	4.46E-01	0.583
		hsa-miR-101-002253	4.63E-01	0.554
		hsa-miR-142-5p-002248	4.79E-01	0.522
		hsa-miR-15b-000390	4.88E-01	0.558
	bottom	hsa-miR-155-002623	8.35E-01	0.514
		hsa-miR-24-000402	8.56E-01	0.536
		hsa-miR-223-002295	8.90E-01	0.5
		hsa-miR-29c-000587	9.18E-01	0.544
		hsa-miR-125b-000449	9.93E-01	0.499

Supplementary Table 2

Bottom subset

(non significative)
from profiling DM1 dataset

Marker0	hsa-miR-125b-000449
Marker1	hsa-miR-155-002623
Marker2	hsa-miR-223-002295
Marker3	hsa-miR-24-000402
Marker4	hsa-miR-29c-000587

CombiROC output:

Symbol	AUC	SE %	SP %	Opt Cutoff
Marker0	0,499	0,708	0,5	0,498
Marker1	0,514	0,875	0,308	0,461
Marker2	0,5	0,542	0,615	0,491
Marker3	0,536	1	0,192	0,457
Marker4	0,544	0,875	0,346	0,464
Combo I	0,502	0,958	0,231	0,423
Combo II	0,502	0,792	0,423	0,507
Combo III	0,486	1	0,077	0,547
Combo IV	0,545	0,75	0,423	0,463
Combo V	0,535	0,75	0,462	0,469
Combo VII	0,572	0,667	0,577	0,483
Combo VI	0,484	0,708	0,538	0,491
Combo VIII	0,532	0,333	0,846	0,505
Combo IX	0,534	0,667	0,5	0,481
Combo X	0,532	0,875	0,385	0,462
Combo XI	0,537	0,875	0,346	0,443
Combo XII	0,55	0,875	0,346	0,459
Combo XIII	0,577	0,792	0,462	0,471
Combo XIV	0,546	0,958	0,308	0,439
Combo XV	0,545	0,875	0,308	0,443
Combo XVI	0,53	0,708	0,462	0,471
Combo XVII	0,534	0,75	0,462	0,463
Combo XVIII	0,572	0,5	0,731	0,495
Combo XIX	0,545	0,667	0,538	0,482
Combo XX	0,538	0,667	0,5	0,48
Combo XXI	0,572	0,917	0,346	0,451
Combo XXII	0,572	0,875	0,385	0,446
Combo XXIII	0,579	0,625	0,654	0,49
Combo XXIV	0,548	0,958	0,308	0,438
Combo XXV	0,454	0,542	0,538	0,486
Combo XXVI	0,585	0,833	0,462	0,46

Middle subset

(non significative)
from profiling DM1 dataset

Marker0	hsa-miR-101-002253
Marker1	hsa-miR-142-5p-002248
Marker2	hsa-miR-145-002278
Marker3	hsa-miR-148a-000470
Marker4	hsa-miR-15b-000390

CombiROC output:

Symbol	AUC	SE %	SP %	Opt Cutoff
Marker0	0,554	0,583	0,615	0,479
Marker1	0,522	0,958	0,269	0,43
Marker2	0,583	0,583	0,654	0,46
Marker3	0,635	0,75	0,654	0,481
Marker4	0,558	0,583	0,731	0,483
Combo I	0,535	1	0,231	0,399
Combo II	0,54	0,583	0,577	0,464
Combo III	0,641	0,708	0,615	0,478
Combo IV	0,587	0,417	0,846	0,504
Combo V	0,487	0,708	0,423	0,516
Combo VI	0,615	0,75	0,577	0,466
Combo VII	0,548	0,958	0,231	0,416
Combo VIII	0,652	0,875	0,462	0,446
Combo IX	0,561	0,833	0,462	0,44
Combo X	0,635	0,75	0,615	0,475
Combo XI	0,452	0,375	0,731	0,46
Combo XII	0,644	0,833	0,577	0,456
Combo XIII	0,457	0,542	0,538	0,489
Combo XIV	0,659	0,875	0,462	0,442
Combo XV	0,585	0,75	0,615	0,472
Combo XVI	0,659	0,708	0,692	0,497
Combo XVII	0,627	0,833	0,423	0,422
Combo XVIII	0,625	0,708	0,577	0,487
Combo XIX	0,625	0,708	0,654	0,489
Combo XX	0,628	0,792	0,5	0,454
Combo XXI	0,652	0,583	0,731	0,534
Combo XXII	0,583	0,458	0,769	0,532
Combo XXIII	0,636	0,792	0,538	0,438
Combo XXIV	0,679	0,5	0,846	0,565
Combo XXV	0,643	0,25	1	0,625
Combo XXVI	0,675	0,667	0,692	0,509

Borderline subset

(non significative)
from profiling DM1 dataset

Marker0	hsa-let-7e-002406
Marker1	hsa-miR-146b-001097
Marker2	hsa-miR-20a-000580
Marker3	hsa-miR-21-000397
Marker4	hsa-miR-27a-000408

CombiROC output:

Symbol	AUC	SE %	SP %	Opt Cutoff
Marker0	0,671	0,625	0,769	0,561
Marker1	0,619	0,458	0,808	0,502
Marker2	0,624	0,458	0,846	0,527
Marker3	0,579	0,5	0,731	0,481
Marker4	0,708	0,75	0,692	0,497
Combo I	0,699	0,792	0,577	0,429
Combo II	0,696	0,667	0,808	0,54
Combo III	0,684	0,625	0,769	0,568
Combo IV	0,702	0,667	0,846	0,573
Combo V	0,647	0,792	0,5	0,404
Combo VI	0,617	0,458	0,808	0,502
Combo VII	0,692	0,875	0,5	0,398
Combo VIII	0,628	0,458	0,808	0,527
Combo IX	0,713	0,75	0,654	0,482
Combo X	0,708	0,75	0,692	0,51
Combo XI	0,702	0,625	0,769	0,493
Combo XII	0,7	0,792	0,577	0,425
Combo XIII	0,723	0,583	0,846	0,542
Combo XIV	0,692	0,708	0,731	0,522
Combo XV	0,702	0,667	0,808	0,565
Combo XVI	0,71	0,625	0,846	0,578
Combo XVII	0,649	0,792	0,5	0,412
Combo XVIII	0,689	0,875	0,462	0,399
Combo XIX	0,713	0,917	0,5	0,362
Combo XX	0,713	0,792	0,615	0,446
Combo XXI	0,704	0,625	0,769	0,492
Combo XXII	0,723	0,625	0,769	0,506
Combo XXIII	0,721	0,625	0,731	0,517
Combo XXIV	0,713	0,708	0,731	0,514
Combo XXV	0,707	0,875	0,538	0,376
Combo XXVI	0,721	0,583	0,808	0,534

Top subset

(significative)
from profiling/screening DM1 dataset

Marker0	hsa-miR-133a-002246
Marker1	hsa-miR-191-002299
Marker2	hsa-miR-193b-002367
Marker3	hsa-miR-574-3p-002349
Marker4	hsa-miR-140-3p-002234

CombiROC output:

**Symbol**	**AUC**	**SE %**	**SP %**	**Opt Cutoff**
Marker0	0,896	0,917	0,731	0,3
Marker1	0,845	0,792	0,808	0,451
Marker2	0,785	0,917	0,615	0,295
Marker3	0,742	0,75	0,692	0,418
Marker4	0,756	0,708	0,731	0,423
Combo I	0,931	0,917	0,846	0,367
Combo II	0,907	0,917	0,769	0,312
Combo III	0,899	0,917	0,769	0,348
Combo IV	0,904	0,917	0,731	0,301
Combo V	0,869	0,917	0,692	0,315
Combo VI	0,849	0,792	0,808	0,388
Combo VII	0,856	0,917	0,692	0,314
Combo VIII	0,803	0,75	0,769	0,456
Combo IX	0,84	0,833	0,808	0,464
Combo X	0,784	0,625	0,885	0,512
Combo XI	0,918	0,917	0,885	0,362
Combo XII	0,931	0,917	0,846	0,367
Combo XIII	0,929	0,917	0,846	0,363
Combo XIV	0,907	0,917	0,769	0,307
Combo XV	0,91	0,833	0,885	0,465
Combo XVI	0,902	0,875	0,808	0,383
Combo XVII	0,87	0,917	0,692	0,316
Combo XVIII	0,873	0,833	0,808	0,394
Combo XIX	0,859	0,833	0,769	0,362
Combo XX	0,84	0,833	0,808	0,466
Combo XXI	0,92	0,917	0,885	0,399
Combo XXII	0,918	0,917	0,885	0,365
Combo XXIII	0,929	0,917	0,846	0,365
Combo XXIV	0,905	0,792	0,885	0,48
Combo XXV	0,875	0,875	0,769	0,384
Combo XXVI	0,92	0,917	0,885	0,399

Top subset

(significative)
from validation DM1 dataset

Marker0	hsa-miR-133a-002246
Marker1	hsa-miR-191-002299
Marker2	hsa-miR-193b-002367
Marker3	hsa-miR-574-3p-002349
Marker4	hsa-miR-140-3p-002234

CombiROC output:

**Symbol**	**AUC**	**SE %**	**SP %**	**Opt Cutoff**
Marker0	0,943	0,889	0,917	0,706
Marker1	0,865	0,75	0,917	0,685
Marker2	0,841	1	0,583	0,285
Marker3	0,907	0,889	0,806	0,658
Marker4	0,908	0,833	0,917	0,716
Combo I	0,924	0,833	0,861	0,67
Combo II	0,927	0,972	0,778	0,443
Combo III	0,92	0,889	0,833	0,689
Combo IV	0,932	0,917	0,833	0,69
Combo V	0,827	1	0,583	0,301
Combo VI	0,905	0,917	0,694	0,467
Combo VII	0,914	0,889	0,806	0,504
Combo VIII	0,91	0,972	0,806	0,337
Combo IX	0,933	0,944	0,861	0,469
Combo X	0,918	0,889	0,861	0,706
Combo XI	0,917	0,972	0,833	0,671
Combo XII	0,924	0,861	0,806	0,609
Combo XIII	0,93	0,889	0,861	0,561
Combo XIV	0,922	0,972	0,806	0,433
Combo XV	0,94	0,917	0,917	0,644
Combo XVI	0,925	0,889	0,861	0,72
Combo XVII	0,91	0,972	0,806	0,31
Combo XVIII	0,93	0,944	0,861	0,566
Combo XIX	0,92	0,861	0,833	0,59
Combo XX	0,942	0,944	0,889	0,537
Combo XXI	0,917	0,944	0,861	0,641
Combo XXII	0,938	0,944	0,917	0,679
Combo XXIII	0,93	0,917	0,806	0,476
Combo XXIV	0,942	0,944	0,889	0,537
Combo XXV	0,938	0,944	0,917	0,65
Combo XXVI	0,938	0,944	0,917	0,646

Data Table 1

Patient.ID	Class	Marker1	Marker2	Marker3	Marker4	Marker5
**AIH1**	A	438	187	197	298	139
**AIH2**	A	345	293	134	523	335
**AIH3**	A	903	392	300	1253	0
**AIH4**	A	552	267	296	666	22
**AIH5**	A	1451	760	498	884	684
**AIH6**	A	497	260	175	640	572
**AIH7**	A	3878	2858	510	1518	348
**AIH8**	A	740	368	266	1391	647
**AIH9**	A	538	412	282	1071	474
**AIH10**	A	1194	1065	442	1530	816
**AIH11**	A	988	817	394	1626	1028
**AIH12**	A	261	153	528	449	237
**AIH13**	A	312	181	58	408	661
**AIH14**	A	499	302	181	1088	568
**AIH15**	A	998	431	75	397	519
**AIH16**	A	6485	3704	8834	2595	1570
**AIH17**	A	8584	2662	13178	3390	2757
**AIH18**	A	1696	1029	369	468	2422
**AIH19**	A	774	505	251	1014	517
**AIH20**	A	433	330	92	1266	743
**AIH21**	A	1207	1501	57	1847	741
**AIH22**	A	225	849	125	330	168
**AIH23**	A	1327	485	366	563	878
**AIH24**	A	76	582	601	115	73
**AIH25**	A	586	256	53	710	74
**AIH26**	A	263	196	124	798	95
**AIH27**	A	146	568	64	125	92
**AIH28**	A	537	620	82	350	53
**AIH29**	A	52	41	588	346	50
**AIH30**	A	882	138	47	601	85
**AIH31**	A	462	304	81	378	208
**AIH32**	A	372	149	0	460	156
**AIH33**	A	1964	875	404	1883	1021
**AIH34**	A	2809	1633	451	1042	608
**AIH35**	A	994	505	303	1699	783
**AIH36**	A	361	185	115	466	520
**AIH37**	A	275	384	45	329	466
**AIH38**	A	947	622	16	236	728
**AIH39**	A	405	133	32	284	358
**AIH40**	A	922	689	68	105	643
**no AIH1**	B	510	28	0	332	0
**no AIH2**	B	205	0	0	332	0
**no AIH3**	B	44	0	0	332	21
**no AIH4**	B	49	38	7	178	48
**no AIH5**	B	26	33	6	131	32
**no AIH6**	B	24	26	8	206	28
**no AIH7**	B	243	373	109	631	477
**no AIH8**	B	99	111	51	193	197
**no AIH9**	B	85	55	71	811	321

**no AIH10**	B	146	108	46	173	171
**no AIH11**	B	0	113	0	0	56
**no AIH12**	B	151	0	0	360	0
**no AIH13**	B	0	0	0	15	21
**no AIH14**	B	0	5	0	147	53
**no AIH15**	B	118	69	13	748	97
**no AIH16**	B	31	29	7	223	34
**no AIH17**	B	72	53	12	376	85
**no AIH18**	B	137	151	20	662	131
**no AIH19**	B	78	58	6	368	49
**no AIH20**	B	193	14	90	703	177
**no AIH21**	B	0	0	0	0	0
**no AIH22**	B	0	20	0	66	31
**no AIH23**	B	351	70	0	478	7
**no AIH24**	B	171	127	74	1202	224
**no AIH25**	B	77	75	26	316	61
**no AIH26**	B	51	45	2	286	44
**no AIH27**	B	64	55	17	333	46
**no AIH28**	B	150	185	42	975	252
**no AIH29**	B	149	140	52	1087	209
**no AIH30**	B	147	103	19	794	98
**no AIH31**	B	86	89	44	427	144
**no AIH32**	B	94	57	18	581	88
**no AIH33**	B	56	41	23	244	49
**no AIH34**	B	10	15	2	121	26
**no AIH35**	B	46	22	0	69	24
**no AIH36**	B	84	65	4	278	77
**no AIH37**	B	42	44	19	306	86
**no AIH38**	B	46	51	24	342	74
**no AIH39**	B	73	39	9	358	41
**no AIH40**	B	83	131	27	1012	124
**no AIH41**	B	0	193	50	382	135
**no AIH42**	B	41	94	64	464	141
**no AIH43**	B	0	77	46	398	85
**no AIH44**	B	98	152	91	469	301
**no AIH45**	B	78	85	10	407	0
**no AIH46**	B	21	16	0	536	16
**no AIH47**	B	44	34	74	266	21
**no AIH48**	B	144	76	63	252	0
**no AIH49**	B	162	249	101	134	0
**no AIH50**	B	100	169	33	1430	0
**no AIH51**	B	52	159	57	681	417
**no AIH52**	B	97	86	64	591	33
**no AIH53**	B	73	80	20	157	51
**no AIH54**	B	162	117	25	749	0
**no AIH55**	B	100	220	37	729	41
**no AIH56**	B	227	174	26	290	0
**no AIH57**	B	136	165	23	315	40
**no AIH58**	B	96	213	47	682	780
**no AIH59**	B	69	59	21	629	245
**no AIH60**	B	74	70	67	138	215
**no AIH61**	B	129	118	44	117	769

**no AIH62**	B	265	173	115	1157	1310
**no AIH63**	B	308	265	290	2464	840
**no AIH64**	B	276	179	203	1848	563
**no AIH65**	B	173	119	194	2245	726
**no AIH66**	B	354	156	0	227	109
**no AIH67**	B	73	25	0	267	68
**no AIH68**	B	111	80	42	384	180
**no AIH69**	B	148	90	54	479	235
**no AIH70**	B	229	367	12	3	595
**no AIH71**	B	388	316	0	0	0
**no AIH72**	B	0	5	0	300	323
**no AIH73**	B	0	66	22	92	0
**no AIH74**	B	0	47	0	337	0
**no AIH75**	B	0	256	42	760	0
**no AIH76**	B	107	61	26	391	0
**no AIH77**	B	174	109	57	428	223
**no AIH78**	B	42	46	13	235	319
**no AIH79**	B	180	146	34	362	249
**no AIH80**	B	79	66	57	378	307
**no AIH81**	B	419	86	51	376	533
**no AIH82**	B	448	133	95	631	122
**no AIH83**	B	0	119	49	399	154
**no AIH84**	B	148	90	67	285	167
**no AIH85**	B	11	155	19	231	0
**no AIH86**	B	165	162	167	912	405
**no AIH87**	B	320	150	88	107	4114
**no AIH88**	B	146	87	111	395	238
**no AIH89**	B	90	102	62	112	267
**no AIH90**	B	257	188	171	644	347
**no AIH91**	B	106	60	63	336	0
**no AIH92**	B	1223	584	640	256	0
**no AIH93**	B	519	287	283	0	0
**no AIH94**	B	327	240	101	102	0
**no AIH95**	B	559	2142	59	279	413
**no AIH96**	B	202	201	29	248	0
**no AIH97**	B	212	88	45	472	0
**no AIH98**	B	426	125	68	99	566
**no AIH99**	B	260	135	105	285	34
**no AIH100**	B	371	257	96	528	75
**no AIH101**	B	254	99	105	332	13
**no AIH102**	B	107	86	121	786	168
**no AIH103**	B	265	209	40	328	10
**no AIH104**	B	201	189	31	559	0
**no AIH105**	B	79	63	38	159	2
**no AIH106**	B	111	34	32	343	13
**no AIH107**	B	130	185	0	577	923
**no AIH108**	B	58	29	11	545	0
**no AIH109**	B	49	56	34	390	0
**no AIH110**	B	158	165	68	227	0
**no AIH111**	B	159	205	25	151	32
**no AIH112**	B	144	155	25	600	0
**no AIH113**	B	111	155	83	179	65

**no AIH114**	B	224	115	27	276	67
**no AIH115**	B	85	85	13	123	48
**no AIH116**	B	362	296	11	176	72
**no AIH117**	B	273	264	122	1418	476
**no AIH118**	B	425	402	86	1702	581
**no AIH119**	B	315	418	0	595	305
**no AIH120**	B	463	379	71	626	313
**no AIH121**	B	94	413	46	400	229
**no AIH122**	B	163	115	285	590	213
**no AIH123**	B	0	0	0	1053	362
**no AIH124**	B	0	0	0	430	26
**no AIH125**	B	83	61	299	476	219
**no AIH126**	B	381	303	4	266	376
**no AIH127**	B	253	106	134	626	189
**no AIH128**	B	571	96	31	487	423
**no AIH129**	B	288	298	0	347	411
**no AIH130**	B	135	149	229	864	600

Data Table 2

Patient.ID	Class	Marker1	Marker2	Marker3	Marker4	Marker5	Marker6
**PCNSL1**	A	10.9	2.8	7	0.1	0.1	3.9
**PCNSL2**	A	7.9	7.2	12.8	0.4	0.3	3.1
**PCNSL3**	A	12.2	8.4	14	0.1	0.1	0.1
**PCNSL4**	A	12.1	3.1	7.6	0.4	0.4	7.1
**PCNSL5**	A	241.1	3.5	11.9	0.3	0.5	0.2
**PCNSL6**	A	69.2	5.8	66.7	0.1	0.1	1.7
**PCNSL7**	A	48.8	8.1	14.8	0.7	0.8	6.9
**PCNSL8**	A	14	11	50.2	0.6	0.4	7.4
**PCNSL9**	A	291.7	6.8	31.1	0.9	1.7	2.9
**PCNSL10**	A	34.3	7.8	15.7	0.2	0.2	1.4
**PCNSL11**	A	14.8	1.4	2.6	0.1	0.1	5.8
**PCNSL12**	A	9.8	3.4	7.6	0.2	0.4	15.1
**PCNSL13**	A	112.9	94.9	128.8	6.5	0.9	89.8
**PCNSL14**	A	64.6	8.7	19.1	0.5	1.1	1.3
**PCNSL15**	A	18.6	10.1	12.2	0.5	0.4	12.5
**PCNSL16**	A	23.5	7.7	18.8	0.6	0.3	8.6
**PCNSL17**	A	239.4	7.3	25.9	0.4	2.4	16
**PCNSL18**	A	13.6	2	7.1	0.3	0.3	5.5
**PCNSL19**	A	10.5	4.7	9.9	0.2	0.3	5.5
**PCNSL20**	A	45.1	2.4	11.4	0.3	0.4	6.8
**PCNSL21**	A	11.3	2.2	7	0.2	0.3	12.9
**PCNSL22**	A	35.7	3.4	12.5	0.3	0.1	1.3
**PCNSL23**	A	38.7	7	21.8	0.3	0.2	4.9
**C1**	B	1.9	1.6	18	0.5	202.8	18.2
**C2**	B	1.8	0.7	6.7	0.1	48.3	14.4
**C3**	B	0.2	0.4	1	0	0	3.2
**C4**	B	0.2	2	3.3	0	0	18.6
**C5**	B	0	0.2	0.7	0.3	24.8	1.3
**C6**	B	0	0.5	1	0	14.6	7
**C7**	B	0.6	0.1	0.6	0.3	0	0.1
**C8**	B	0.3	0.9	1.7	0	0.1	9.3
**C9**	B	0.2	1.7	1.8	0.3	0.1	1.6
**C10**	B	0.2	0.2	17.4	0.2	0.1	0.7
**C11**	B	0.1	3.5	6.4	1.7	0.5	1.7
**C12**	B	1.5	0.3	1.1	9.7	0.1	14.2
**C13**	B	1.1	0.3	0.7	0.5	0	2.5
**C14**	B	0.9	0.2	0.6	8.5	0	12.2
**C15**	B	1.8	0.3	0.9	0.4	0.1	8.4
**C16**	B	2.8	1.3	2.9	2.5	0.4	3.4
**C17**	B	23.8	0.1	0.9	1.4	0.2	0.7
**C18**	B	1.4	0	1	0.9	6.9	2.4
**C19**	B	0.3	0	0.7	0	0.3	0.8
**C20**	B	0	5.1	0.2	0	0.2	1.8
**C21**	B	2.4	0	0.8	0	2.4	4.4
**C22**	B	5.8	0.1	0.3	0	0.1	0.5
**C23**	B	14.8	0.1	0.5	0.3	0.2	0.8
**C24**	B	13.2	0.3	1.1	0	4.5	0.4
**C25**	B	13.9	0.3	2.7	0	0.5	0.8
**C26**	B	9	0.1	0.8	0	0.2	19.1

**C27**	B	10.4	0.1	0.3	0	0	0.3
**C28**	B	4.1	0	0.4	0	0.1	1.6
**C29**	B	3.9	0	0.4	0	0.1	2.2
**C30**	B	5.4	0	0.4	0	0.2	0.6

COMBIROC'S TUTORIAL.

A web app for guided and interactive generation of multimarker panels (*www.combiroc.eu*)

Overview of the CombiROC workflow.

CombiROC delivers a simple workflow to help researchers in selecting the optimal combination(s) of markers through a simple analytical method based on the introduction of a double filter scoring. **Figure 1,** left, illustrates the general workflow of the application: after uploading multi markers profiling data in text format, users are offered a choice of simple data viewing with plotting and optional data processing methods. Users can define the stringency of their test (i.e. the signal cutoff and minimum number of positive features). Then, thresholds on sensitivity (SE) and specificity (SP) can be freely explored and interactively adjusted graphically observing how many markers' combinations survive the cutoffs; finally, the best combinations can be chosen and their ROC curves automatically generated. Users can review results and download combinatorial analyses results and ROC curves.

CombiROC's analytical approach is based on sensitivity and specificity filters, interpreted in terms of recognition frequency, optimizing the number of potentially interesting panels rising from a previous combinatorial analysis step. CombiROC does not take for granted a default algorithm-driven marker threshold, but it allows users to interactively choose the thresholds according to their requirements: in doing so it dramatically reduces the computational burden for the subsequent analytical steps. CombiROC also makes the analysis of biomarkers panels of diverse nature easier, lowering false negative rate given by fixed thresholds.

Paragraphs of this tutorial are ordered following the structure of the application's main menu (**Figure 1**, right).

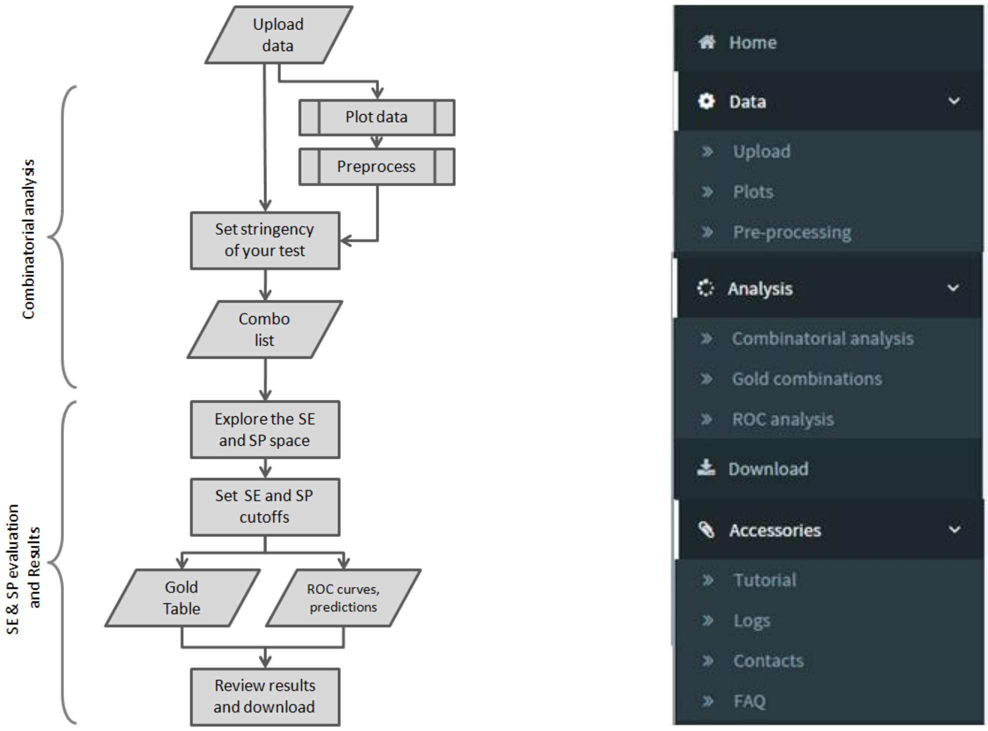

FIGURE 1

DATA

UPLOAD

Data can be uploaded to the CombiROC application. Before being uploaded in the application, data need to be correctly formatted as text files (csv, tab or semicolon separated). Make sure you are using the English locale for decimal separators (use the dot "." to separate decimals in numerical fields).

You can prepare your data in your favorite spreadsheet software or application as long as it contains (see **Figure 2**):

- in the first column, the samples IDs (*i.e.* patients number) ,
- in the second column, the class category (*i.e.* disease and healthy; marked "A" and "B")
- from the third column onward, the data values (*i.e.* detection levels)

The first row can contain a header. An example is given in **Figure 2**, and a preformatted demo file can be also downloaded.

- the header of the first column must be "Sample_ID"
- the header of the second column must be "Class"
- the header of columns from the third onward must be "Marker#", where "#" is a progressive integer (*i.e.* Marker1, Marker2, Marker3, …).

In the second column, dedicated to the classes/categories of samples (i.e. healthy and diseases; treated and untreated) an arbitrary number of classes are accepted in the file but *the application will consider for the analyses only the first 2 classes for pairwise comparisons*. Thus, for clarity we recommend to limit in the uploaded file the number of classes to 2, tagged with "A" and "B".

Once your data are correctly formatted you can upload them using the "Data / Upload" link in the main menu on the left: in the "Enter data" widget select the "Upload file" option then select the file from your workstation. In case it's not automatically recognized, you can indicate the presence or absence of the header and specify the separator used (comma, semicolon or tab). From the very same menu you are also offered the possibility to use the pre-loaded demo data choosing the "Load demo data" options.

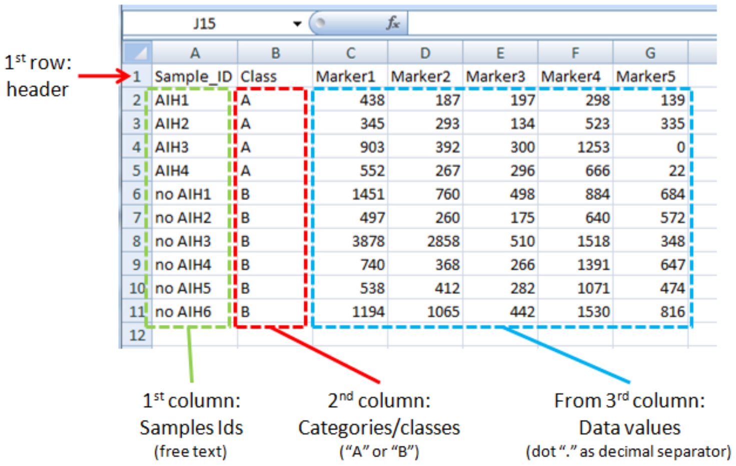

	A	B	C	D	E	F	G
1	Sample_ID	Class	Marker1	Marker2	Marker3	Marker4	Marker5
2	AIH1	A	438	187	197	298	139
3	AIH2	A	345	293	134	523	335
4	AIH3	A	903	392	300	1253	0
5	AIH4	A	552	267	296	666	22
6	no AIH1	B	1451	760	498	884	684
7	no AIH2	B	497	260	175	640	572
8	no AIH3	B	3878	2858	510	1518	348
9	no AIH4	B	740	368	266	1391	647
10	no AIH5	B	538	412	282	1071	474
11	no AIH6	B	1194	1065	442	1530	816
12							

FIGURE 2

Immediately after loading the data, they will be visualized in a tabular form in the “Table” widget on the right; if the data are correctly formatted you will be able to see the header and data as they appear in the original file. Only the first ten entries (rows) are displayed by default but you can adjust this number with the upper left selection in the Table widget.

The displayed rows can be copied, downloaded as csv, or as pdf clicking on the “Copy”, “CSV” or “PDF” buttons respectively, on the upper right corner of the widget. Please note that only entries displayed on screen will be downloaded or printed, so if you want the entire file to be downloaded make sure to select all entries with the “Show ALL entries” toggle selection on the left.

The widget “Details of uploaded dataset” will summarize some details of your data: the number of samples, markers and categories, the data value’s type and the presence of missing values. If errors are detected a warning will be displayed in this widget.

PLOTS

The “Plots” page of CombiROC automatically displays two types of plots and statistics overview of the uploaded data. Upon clicking to the Data / Plots menu, ancillary options become active on the lower bottom of the main menu: they are “Display” , used to choose from two different type of plots (box plot and marker profile plot), and “Options” (available for the box plot only) used to change data, display and label options of the box plot.

Among the options that can be changed for the box plot type are the whisker type, the color of boxes, the orientation, height and width of the plot. The Y-axis ranges of the two classes’ plots are scaled individually by default, according to minimum and maximum values in the dataset. If you need to have both box plot panels in the same range you can adjust this range in the “Adjust Y-axis range” field typing it in the format “0,1000” (lower value - comma - upper value). Beware that adjusting Y-axis range the value extremes could be not visualized. Finally, labels can be edited with custom text and font size.

The box plot is used to visualize the distribution of single markers values across the samples (i.e. patients). Upon loading the demo data two box plots are obtained colored in red-pink for Class A and blue for Class B; markers are visualized in each class and their distributions observed.

In the box plot page is also visible the “Box plot statistics” tables for both classes: they include distribution parameters which allow the user to choose the best cutoff values for the subsequent step (combinatorial analysis).

The marker plot displays the signal intensity of each single sample for each marker. Select the marker you want to display from the drop down menu on the left, click on “plot graph” and the profile plot will be displayed. You then can hover over data points to reveal single samples details.

PRE-PROCESSING (OPTIONAL)

In this page data can be processed with a few transformations if they need to be reshaped. From the drop down menu you can choose among a data transformation (log2 transformation), and two methods of scaling (unit variance scaling; pareto scaling).

The “unit variance scaling”, also known as autoscaling is commonly applied and uses the standard deviation as the scaling factor; in the “pareto scaling” the square root of the standard deviation is used as the scaling factor instead.

On the right side of the page the transformed and/or scaled data are displayed in tabular form. As for the other tables visualized in the application, only the first ten entries (rows) are displayed by default but you can adjust this number with the upper left selection in the Table widget.

PLEASE NOTE: *CombiROC is neither a transformation nor a data visualization tool: the steps "Plots" and "Pre-processing" are not strictly necessary for the completion of the analysis. The "Plot" function and the optional transformation tools are meant to allow users to look their data's structure, but data themselves should be correctly formatted* ***before*** *being uploaded to CombiROC.*

ANALYSIS

COMBINATORIAL ANALYSIS

The Combinatorial Analysis, also called combinatorics, is a branch of mathematics concerned with the theory of enumeration, or combinations and permutations, in order to solve problems about the possibility of constructing arrangements of objects which satisfy specified conditions.

In the page “Analysis / Combinatorial Analysis” you will find tools to obtain all possible markers combinations and choose the best one, i.e. the one with the higher response. Three main widgets “**Graphics**”, “**Mathematical details**” and “**Combo List**” are describe below.

GRAPHICS

In the “Graphics” widget you can set, according to the specific nature of your experiment (the test), the cutoff above which the features’ values are considered positive (the "*test's signal cutoff*"); you can also insert the minimum number of positive features that need to reach the previously set cutoff.

As an example, upon loading the “Demo data (proteomics)” provided by the application you will find the pre-set cutoff value of “450” (e.g. Fluorescence Intensities, representing the mean value of buffer control class plus three times the standard deviation). The minimum number of positive features is, for these demo data, pre-set to “1”, *i.e.* with the minimum stringency, which means that at least 1 marker must reach the value of 450.

Once you have set the proper thresholds click on the "*Distributions*" button to visualize in an histogram graph the distributions of *Sensitivity* and *Specificity* of the combinations satisfying the set cutoffs (**Figure 3**): *Sensitivity* (SE, blue bars) is defined as the true positive rate in percentage of your sample. *Specificity* (SP, black bars) is defined as the true negative rate in control class in percentage. In x-axis is shown the number of each positive feature as frequency (left wise blue bars for SE intervals, right wise black bars for SP intervals) while in y-axis the SE and SP distributions intervals in percent. You can hover over bars to see values.

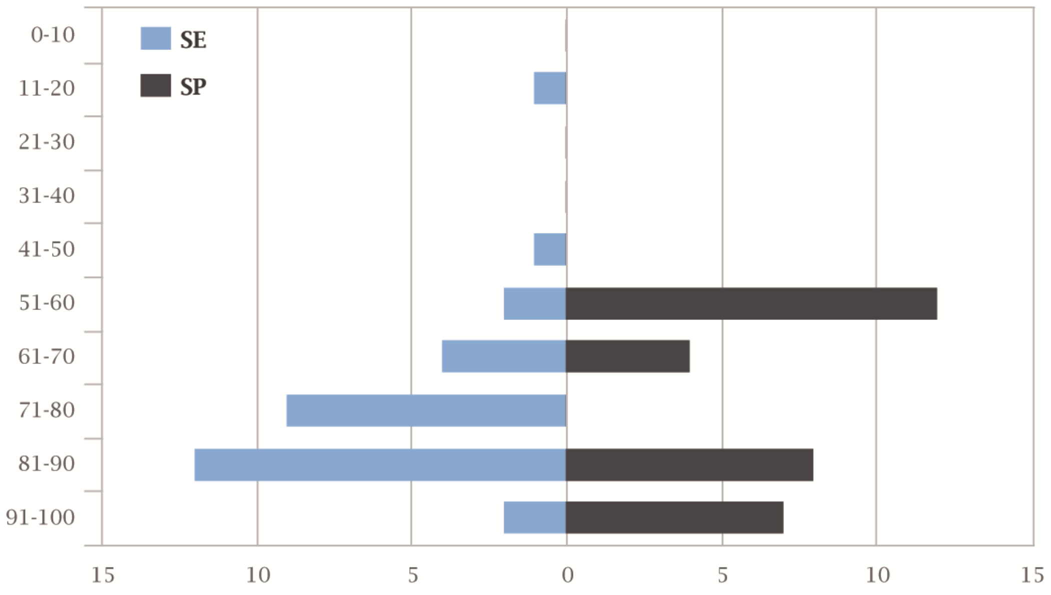

FIGURE 3

This histogram plot helps the user to evaluate the intervals from which the best SP/SE values will be chosen, and on which markers’ “*Gold Combinations*” will be calculated in further steps of the analysis. In the specific “Demo data (proteomics)” example, to which the plot in **Figure 3** refers, the graph shows that most markers’ combinations have

sensitivity higher than 40%, with a peak of 12 combinations in the 81-90% sensitivity range; for the specificity distributions all combinations have SP higher than 50% and a substantial number of them are above 80%. Any evaluation and choice at this point is strictly dependent on the specific nature of the experiment that generated the data and on the aim of the user: using the demo data as an example the preloaded values of sensitivity >40% and specificity >80% can be found, since they may serve the purpose of a usable tradeoff with the data at hand.

Once these "hard" thresholds are set, further browsing and evaluation of sensitivity and specificity values can be done in the subsequent "Gold combinations" section.

MATHEMATICAL DETAILS

In this widget is shown the formula used for the combinatorial analysis:

$$C_{tot} = \sum_{k=0}^{N} C(N,k) = 2^N - 1$$

N= the total number of items; k= the desiderate number of components in the combination. For more theoretical details see: Introductory Combinatorics (5th Edition), Brualdi RA.

COMBO LIST

The table in the "Combo List" widget shows a numerical overview of the sensitivity and specificity of combination of markers thereof according to the thresholds set in the "Graphic" widget. In the "Demo data (proteomics)" example the table shows the sensitivity and specificity values of 31 features, a list that includes each marker and all possible combinations generated using the pre-set threshold (at least 1 feature with ≥ 450 detection, i.e. at least 1 feature with "positive" value).

GOLD COMBINATIONS

Once the hard thresholds (*i.e.* cutoffs on detection value and minimal number of markers) have been set, the array of obtained markers' combinations ("Combos") can be more deeply evaluated in order to select only the few (the Gold ones) that satisfy a minimal SP and SE.

EXPLORE SE AND SP VALUES / GOLD COMBINATION BUBBLE PLOT

In this section two sliders are available to explore the SE and SP ranges. On the Bubble chart on the right of the page sensitivity (Y axis) and specificity (X axis) of all the marker combinations are automatically plotted ; the size of the bubbles is proportional to the number of markers in the combo, the bigger the bubble, the more the markers. Combinations that do not bypass the SE & SP thresholds set with the sliders on the left are depicted as blue bubbles (the "under the thresholds" combos), otherwise the bubbles are yellow (the "Gold" combos). To start off, you can move the sensitivity and specificity sliders and observe how many bubbles (=markers combos) remain yellow at higher sensitivity and/or specificity values.

GOLD TABLE

Once you reach a reasonable tradeoff between SE, SP and number of combos to go further in the analysis you need to confirm SE and SP values in the "Gold Table" section, in the lower half of the page (you'll notice that SE and SP values set with the sliders are automatically reported in the "Gold Table" section below; for the "Demo data (proteomics)" values of 40% Sensitivity and 80% Specificity are suggested and preloaded). Click on "Submit" and a table detailing - and naming - the selected combinations will be displayed in the lower right of the page (**Figure 4**). This table lists each single marker and/or combination of markers that have been selected from the values of *Specificity* and *Sensitivity* used. Using the "Demo data (proteomics)", and the preloaded SP and SE values (see also discussion in the previous section "Combinatorial Analysis"), 14 combinations out of the 31 in the original input list are selected and

consequently displayed in the gold table: this table displays the percentages of Specificity and Sensitivity corresponding to each marker or combination. As other tables in the application, this one can be copied and downloaded as a csv or pdf file.

Show 25 entries Search: Copy CSV PDF

Combinations	Symbol	SE%_disease	SP%_control
Marker1	Marker1	65	95
Marker2	Marker2	48	98
Marker5	Marker5	58	88
Marker1-Marker2	Combo I	72	95
Marker1-Marker3	Combo II	72	95
Marker1-Marker5	Combo III	75	84
Marker2-Marker3	Combo IV	52	98
Marker2-Marker5	Combo V	70	87
Marker3-Marker5	Combo VI	68	88
Marker1-Marker2-Marker3	Combo VII	78	95
Marker1-Marker2-Marker5	Combo VIII	82	84
Marker1-Marker3-Marker5	Combo IX	82	84
Marker2-Marker3-Marker5	Combo X	75	87
Marker1-Marker2-Marker3-Marker5	Combo XI	88	84

FIGURE 4

ROC ANALYSIS

After having set all the thresholds on your dataset and obtained a number of "Gold combinations" of markers, then you finally need to see how these combinations perform. Receiver operating characteristic (ROC) curves are used in medicine to determine a cutoff value for a clinical test. When creating a diagnostic test, a ROC curve helps us visualize and understand the tradeoff between high Sensitivity and high Specificity when discriminating between clinically "*normal*" and clinically "*abnormal*" laboratory values.

SELECT COMBOS / RESULTS

First, you have to select from the drop down menu the single combination to visualize the corresponding ROC curves. If you want to directly compare multiple markers or combinations in the same plot thick the "Check to plot multiple curves" mark (see further). The names of markers and combinations in the drop down menu ("Marker#" if single markers or "Combo" followed by roman numeral) are the same as those reported in the "Gold table" in the previous page of the workflow.

Once you select a single marker or combo from the dropdown, the ROC curve, Predictions and Performance Analysis are automatically calculated and visualized.

ROC CURVES

The ROC curve (**Figure 5**) is a graph of "sensitivity" (y-axis) *versus* "1 – specificity" (x-axis). Large "y" values on the ROC curve plot correspond to higher sensitivity, while small "x" values correspond to higher specificity. The shape of the curve depicts the combinatorial variation of these two important parameters. Below the ROC curve you will be able to see the AUC (Area Under the Curve), SE, SP and optimal cutoff numerical values of the analyzed combo in tabular form.

The results are reported in fractions from 0 to 1. A diagonal line of identity is reported such as the point of optimal cut-off. However, you can choose whether to display it or not, just by clicking on it.

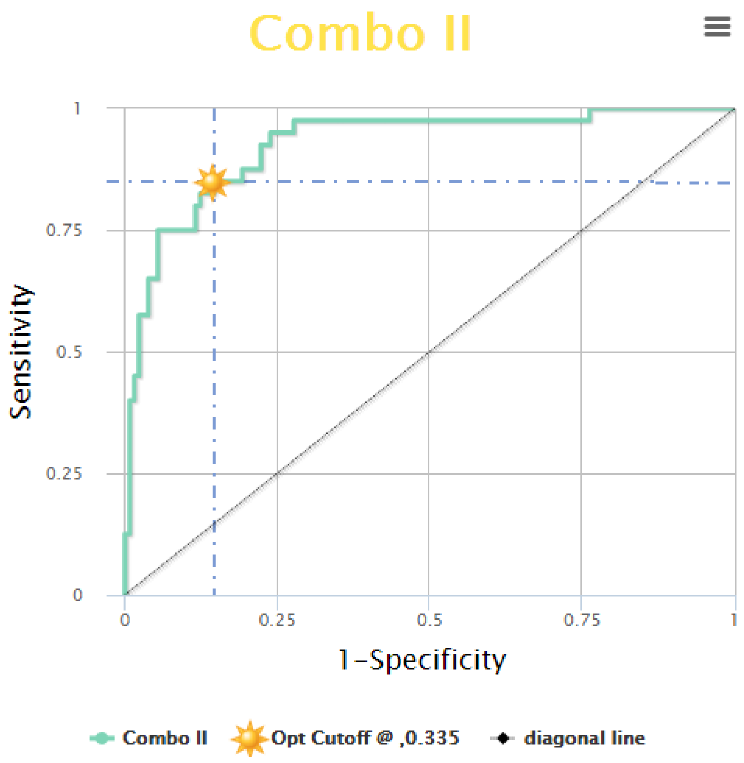

FIGURE 5

Figure 5 shows the ROC curve of "Combo II" obtained using the "Demo data (proteomincs)". A table for each single marker or combo that have been selected from the marker dialog, will also appear below the plot, showing the values of Area Under Curve, Sensitivity, Specificity (in percentage) and Optimal cut-off. As other tables/figures in the application, this one can be copied and downloaded as a csv or pdf file.

PREDICTIONS

The "*Predictions*" section of the page displays a violin plot and a pie chart.

The violin plot is a combination of a box plot and a kernel density plot, showing the "probability density" of the data at different values. Prediction probabilities are plotted for both classes (class A and B, disease/healthy, treated/untreated) according to the previously obtained optimal cutoff. The four possible categories are then visualized: False Negative (FN); False Positives (FP); True Negative (TN) and True Positive (TP). This plot helps to visualize the proportion of samples falling in the four possible quadrants, especially in those of the TN and TP predicted categories, in order to evaluate the goodness of the underlying marker or combination.

The pie chart shows the very same information in a different way. In this plot can be easily visualized which fraction of false predictions (false positive or false negative) there are in each class (class A/B, disease/healthy) as opposed on how big is the fraction of true predictions and inside the total fraction of markers in each class. Obviously the smaller the false predictions fractions, the better performing is the marker or the combination.

PERFORMANCE ANALYSIS

In the lower section of the page the same ROC curve of the selected combo is overlaid with the corresponding Cross Validation (CV) in order to evaluate its performance. The table below this last plot reports the accuracy (ACC) and error rate of the whole cohort and 10-fold CV, as well as the corresponding sensitivity (SE), specificity (SP) and Area Under the Curve value (AUC).

PLOTTING MULTIPLE CURVES

If you want to visualize and compare ROC curves of multiple markers and combinations among those selected in the Gold table you need to check the “Check to plot multiple curves” tick mark in the “Select Combos” widget at the top (upper left) of the page.

In the drop down selection menu you will be able to choose, one after the other, all the single markers and/or combos in the gold table that you want to compare: a graph of overlaid ROC curves will be automatically displayed in a single plot. Below the graph SE, SP and AUC for each curve will be reported in a tabular form. As other tables/figures in the application, this one can be copied and downloaded as a csv or pdf file.

Please note that prediction and performance analyses as described before are available for single markers or combinations only, not when multiple markers/combos are compared in the same ROC curve plot.

PERMUTATION TEST

In order to test the statistical significance of AUC value you can hit the "Check to perform permutation test (single curves only)" tick mark in the "Select Combos" widget at the top (upper left) of the page. In the drop down selection menu you will be able to choose a marker (single markers or combos) in the gold table that you want to verify and a graph will be displayed. This plot shows the density plot of the AUC values in the analysis of 500 permutation test and the grey line the real AUC value. The table below the plot reports the accuracy (ACC) and error rate of the whole cohort and the permutated models, as well as the corresponding sensitivity (SE), specificity (SP) and Area Under Curve (AUC).

DOWNLOAD

In this section you can download the tabular file of the “demo data” and a printable pdf file of the tutorial.

ACCESSORIES

TUTORIAL

This section describe how to use CombiROC step by step. Real dataset is used as example as “Demo data (proteomics)”. The demo dataset is obtained from Mazzara et al. 2015, PLoS One 10(9):e0137927 and can also be downloaded from the “Download” section of the application.

Other "Demo data (transcriptomics)" available on the CombiROC website are obtained from Baraniskin et al. 2011, Blood 117(11):3140-6.

LOGS

This section contains the version history of the CombiROC web application. All relevant changes and upgrades will be reported here in the future.

CONTACTS

This application was created by the Protein Microarray and Bioinformatics units at Istituto Nazionale Genetica Molecolare “Romeo ed Enrica Invernizzi” (INGM), Milan, Italy.

If you have questions or comments, please contact Saveria Mazzara (mazzara[at]ingm.org). This application was implemented using Shiny (for web interface) and other R packages (for data manipulation).

FAQ

In this section frequently asked questions on the application are reported.
